# *Ijuhya vitellina* sp. nov., a novel source for chaetoglobosin A, is a destructive parasite of the cereal cyst nematode *Heterodera filipjevi*

**DOI:** 10.1371/journal.pone.0180032

**Published:** 2017-07-12

**Authors:** Samad Ashrafi, Soleiman Helaly, Hans-Josef Schroers, Marc Stadler, Katja R. Richert-Poeggeler, Abdelfattah A. Dababat, Wolfgang Maier

**Affiliations:** 1 Institute for Epidemiology and Pathogen Diagnostics, Julius Kühn-Institut (JKI)—Federal Research Centre for Cultivated Plants, Braunschweig, Germany; 2 Department of Ecological Plant Protection, Faculty of Organic Agricultural Sciences, University of Kassel, Witzenhausen, Germany; 3 Department Microbial Drugs, Helmholtz Centre for Infection Research GmbH (HZI), Braunschweig, Germany; 4 Department of Chemistry, Faculty of Science, Aswan University, Aswan, Egypt; 5 Agricultural Institute of Slovenia, Ljubljana, Slovenia; 6 CIMMYT (International Maize and Wheat Improvement Centre), P.K.39 06511 Emek, Ankara, Turkey; Universita degli Studi di Pisa, ITALY

## Abstract

Cyst nematodes are globally important pathogens in agriculture. Their sedentary lifestyle and long-term association with the roots of host plants render cyst nematodes especially good targets for attack by parasitic fungi. In this context fungi were specifically isolated from nematode eggs of the cereal cyst nematode *Heterodera filipjevi*. Here, *Ijuhya vitellina* (Ascomycota, Hypocreales, Bionectriaceae), encountered in wheat fields in Turkey, is newly described on the basis of phylogenetic analyses, morphological characters and life-style related inferences. The species destructively parasitises eggs inside cysts of *H*. *filipjevi*. The parasitism was reproduced in *in vitro* studies. Infected eggs were found to harbour microsclerotia produced by *I*. *vitellina* that resemble long-term survival structures also known from other ascomycetes. Microsclerotia were also formed by this species in pure cultures obtained from both, solitarily isolated infected eggs obtained from fields and artificially infected eggs. Hyphae penetrating the eggshell colonised the interior of eggs and became transformed into multicellular, chlamydospore-like structures that developed into microsclerotia. When isolated on artificial media, microsclerotia germinated to produce multiple emerging hyphae. The specific nature of morphological structures produced by *I*. *vitellina* inside nematode eggs is interpreted as a unique mode of interaction allowing long-term survival of the fungus inside nematode cysts that are known to survive periods of drought or other harsh environmental conditions. Generic classification of the new species is based on molecular phylogenetic inferences using five different gene regions. *I*. *vitellina* is the only species of the genus known to parasitise nematodes and produce microsclerotia. Metabolomic analyses revealed that within the *Ijuhya* species studied here, only *I*. *vitellina* produces chaetoglobosin A and its derivate 19-*O*-acetylchaetoglobosin A. Nematicidal and nematode-inhibiting activities of these compounds have been demonstrated suggesting that the production of these compounds may represent an adaptation to nematode parasitism.

## Introduction

Cyst nematodes are attacked by several fungal species. The first report on cyst-parasitic fungi dates back to 1877, when Julius Kühn described a *Tarichium* species, today known as *Catenaria auxiliaris* (Kuehn) Tribe, as a parasite of the sugar beet nematode *Heterodera schachtii* Schmidt [1]. A diverse group of fungi has since then been described as associates of cyst nematodes [2]. Examples include *Ilyonectria destructans* (Zinssm.) Rossman, L. Lombard & Crous [3], *Pochonia chlamydosporia* (Goddard) Zare & W. Gams (both Ascomycota, Hypocreales) and *Nematophthora gynophila* Kerry & D.H. Crump (Stramenopiles) [4, 5] that were described as parasites of *Heterodera avenae* Wollenweber. Kerry [6] also demonstrated that all these species contribute to the natural suppression of nematode populations of *H*. *avenae*. Similar nematode suppressive effects were also reported from different geographical regions [7–10]. These observations have increased further attention to investigate the association of fungi with cyst nematodes and their biocontrol potential.

The destructive parasitism on nematodes is in some cases linked with the production of biologically active fungal secondary metabolites [11–13] including nematicidal compounds that display various anthelmintic effects [14]. Chaetoglobosins of the cytochalasan family can have cytotoxic and inhibitory activities [15, 16] and affect insects and nematodes [17–21]. In the past decade, the use of synthetic chemical nematicides has either been reduced significantly or they were banned completely for posing health and environmental risks. Therefore, environmentally friendly and biologically effective alternatives for the control of nematode plant pests are urgently needed. They may consist in the application of whole organisms or in biologically active fungal compounds only [12, 13, 22–26].

In this context, experimental fields of the International Maize and Wheat Improvement Centre (CIMMYT) in Turkey were screened for antagonistic fungi associated with the cereal cyst nematode *Heterodera filipjevi*. We report here on the discovery of a new species from the hypocrealean Bionectriaceae and describe its unique destructive parasitism on eggs of *H*. *filipjevi*. Potentially involved secondary metabolites produced by the fungus were isolated, structurally elucidated and their biological activity against nematodes was tested.

## Material and methods

### Nematode collection and materials examined

Naturally nematode-infested experimental fields of CIMMYT located in two different regions in the Central Anatolian Plateau of Turkey including Yozgat (39° 08ʼ N, 34° 10ʼ E; altitude, 985 m) and Haymana (39° 25ʼ 52ʼ N, 39° 29ʼ 44ʼ E; altitude, 1259 m) were sampled at crop maturity in 2013. Nematode cysts were extracted from rhizosphere and roots of nematode susceptible and resistant wheat varieties using the modified flotation decanting method [27] and handpicked from the extracted suspensions under a Leitz dissecting microscope. Cysts were stored in 1.5 mL microtubes at 4°C either in dry condition or in water for further experiments. Reference strains required for taxonomic and phylogenetic inferences were obtained from the Westerdijk Fungal Biodiversity Institute (Utrecht, The Netherlands).

### Ethics statement

Both the sampling sites and the nematode species were not protected and thus no permits were required.

### Fungal isolation from field collected samples

Homogenously brown and visibly emptied cysts of *H*. *filipjevi* were separated from hereafter called symptomatic cysts showing unusual discolourations or fungal colonization. Under a laminar flow hood, symptomatic cysts were surface sterilized in 0.5% sodium hypochlorite (NaOCl) for 10 min and rinsed six times with sterile deionised water (SDW). The sterilizing effect of NaOCl was evaluated. For this, individual cysts were imprinted into potato dextrose agar medium (PDA; Merck, Germany) using a sterile forceps, and immediately transferred to new PDA plates. The control plates were incubated at room temperature and regularly monitored for contaminants for four weeks to exclude not-successfully surface-sterilised cysts from further analyses. Using a sterile forceps and an insect needle, transferred cysts were separately cut open on the fresh agar medium and eggs were dispersed on the agar plate. Cyst debris, *i*.*e*. particles of the cyst wall, was discarded and nematode eggs showing symptoms of fungal infections were rolled gently on agar surface to remove potentially occurring contaminating fungal propagules or hyphae adhering to the surface of eggs. Eggs were then transferred to a sterile watch glass containing SDW. When settled, eggs were rinsed six times by removing and replacing the majority of SDW. Eggs were then surface sterilized in 0.5% NaOCl for 2 min. The disinfection solution was removed and the eggs were washed up to six times with SDW as described above.

Surface-sterilised eggs of each individual cyst were then transferred to a new agar plate using a pipette and divided in two aliquots. Eggs from one portion were individually picked up and placed on PDA amended with penicillin G (240 mg/L) and streptomycin sulphate (200 mg/L) (PDA+). Plates were incubated at room temperature and monitored regularly. Each agar plate received a maximum of 4 individually placed single eggs. Fungi emerging from these eggs were identified using morphological and molecular phylogenetic methods. In addition they were used for studying fungal-nematode interactions *in vitro*. For long-term maintenance, representative fungal isolates were stored cryogenically at -140°C. Individual eggs from the second portion were directly transferred into 1.5 mL microtubes for culture-independent identification. The results of species identifications obtained from the culture-independent method were then compared to the results from the culture-dependent method. By doing so, additional evidence was gathered to prove that the fungi isolated by culturing techniques indeed colonised the nematode eggs.

### Pure culture based studies

Sporulating structures were assessed after the isolates of the here studied fungal species were inoculated on corn meal agar (CMA; Fluka), oatmeal agar (OA; 30 g oatmeal, 18 g agar-agar, 1L deionised water), synthetic nutrient-poor agar (SNA; [28]), malt extract agar (MEA; Carl Roth), yeast malt agar (YMA; [29]), PDA and one third strength PDA (PDA 1/3). Strains were also inoculated on PDA, CMA and SNA supplemented with sterile pieces of filter paper, carnation leaf pieces, or wheat straw. Cultures were incubated for up to 12 months at room temperature, as well as at 10, 15, 20, 25, and 30°C in dark or under different light regimes including ambient lighting, or 12h /12 h cycles of light/darkness or black light/darkness. Growth rates at various temperatures were determined by inoculating cylindrical agar plugs, 4–5 mm diam, excised from the margin of PDA cultures onto PDA (Difco). Colour changes of fungal structures formed in culture were checked in 3% watery solution of potassium hydroxide (KOH). Colour codes used in the description were determined according to Kornerup and Wanscher [30].

### Pathogenicity test and Koch’s postulates

The pathogenicity of the here studied fungal species was tested *in vitro* against cysts and eggs of *H*. *filipjevi* multiplied on wheat plants grown in steamed substrates in the greenhouse. Cysts were extracted and surface-sterilised for the experiments as described above. Three independently isolated strains of the fungus were sub-cultured on PDA+ and incubated for 3 months in 10 replicates. Ten surface-sterilised healthy cysts were then placed on top of each of the colonies. Plates were incubated at room temperature and cysts were monitored at regular intervals for fungal infection. Similar experiments were also done with surface sterilized eggs obtained from healthy cysts. To ensure that there is no contamination, eggs were individually placed on PDA and incubated at ambient temperature. Under a laminar flow hood, eggs not showing any contamination after 2 d of incubation, were individually placed on top or at the edge of 2-month-old PDA+, PDA1/3+ and SNA+ cultures. Plates were incubated at room temperature and eggs were monitored daily.

The process of fungal colonisation of eggs of *H*. *filipjevi* was also studied in modified slide culture experiments [31]. Single microsclerotia formed by the studied fungus (described below) were placed as inoculum in the centre of agar blocks (15×15×2 mm), and up to 20 nematode eggs were placed in their vicinity. Inoculated agar blocks were covered with sterile cover slips and slides were incubated in moist glass chambers at room temperature. Developing structures were monitored and microscopically photographed regularly.

### Light and scanning electron microscopy

Nematode and fungal structures were examined and photographed with a Zeiss Axioskop 2 plus compound microscope and an Olympus SZX 12 stereo microscope equipped with a Jenoptik ProgRes® digital camera supported with CapturePro 2.8 software (Jenoptik, Jena, Germany). Eggs and fungal structures were mounted in water. Cysts were photographed in a square cavity dish in water. All microscopic specimens were studied using Differential Interference Contrast (DIC) optics. Measurements are given as minimum–maximum × minimum–maximum with arithmetical means placed in brackets, followed by the number of measurements (n).

For SEM, fungal structures of interest were either picked directly from the surface of colonies or collected after dissolving a small piece of agar in an agarose dissolving buffer (Zymo Research Corp., Irvine, California, USA). Structures were washed with SDW and placed on non-conductive double-sided adhesive tape on aluminium stubs. Samples were photographed using a FEI Quanta 250 scanning electron microscope (Hillsboro, Oregon, USA) at 12.5 kV in low vacuum. Images were adjusted in brightness and contrast using Adobe Photoshop software CS 5.1.

### DNA extraction

Fungal mycelium was obtained from PDA and transferred to 1.5 mL microtubes. Genomic DNA was extracted with a CTAB-based method [32]. Cells were disrupted by grinding using sterile micro-pestles and then lysed in 800 μl CTAB buffer at 65°C for 1 h and 300 rpm. Removal of proteins and precipitation was achieved in two steps by adding 600 μl chloroform and 350 μl isopropanol. Polar fractions were retrieved through centrifugation. DNA pellets were washed twice with 70% ethanol, re-suspended in molecular grade water or elution buffer, and stored at -20°C.

Infected single eggs were transferred to 1.5 mL Eppendorf microtubes containing 5 μl SDW. Approximately 40 mg sterile silica sand and four 1 mm sterile steal beads were added. The samples were incubated in a laminar flow to evaporate the remaining water. Each sample was then frozen in liquid nitrogen and then disrupted in a tissue lyser (Qiagen TissueLyser LT, Hilden, Germany) at 50 Hz for 2 min. Freezing and disruption steps were repeated three times. DNA was then extracted and purified with the Qiagen DNeasy Plant Mini kit following the manufacturer’s instructions.

### PCR amplification and sequencing

Two domains of the nuclear rDNA gene cluster including the internal transcribed spacers with the 5.8S rDNA gene (ITS) and the 5’ end of the nuclear large subunit ribosomal RNA gene (LSU) were amplified with primers V9G [33] and LR5 [34] and sequenced with primers ITS1F [35], ITS4 [36], LR0R [37], and LR5. Three partial protein-encoding genes were amplified and sequenced including the RNA polymerase II largest subunit 1 (*rpb* 1), actin (*act*), and β-tubulin (*ß-tub*) genes. The primers cRPB1af and RPB1cr [38] were used for amplification and sequencing of *rpb1*; Tact1f and Tact2r [39] were used for *act*, and T1 and T22 for *ß-tub*. For sequencing *ß-tub*, primer T222 and T224 [40] were used as additional internal sequence primers.

All PCR reactions were performed in a final volume of 50 μl containing 1 μl of template DNA and 49 μl of PCR master mix including 5 μl of 10× TrueStart, (NH_4_)_2_SO_4_ amended *Taq* Buffer (Thermo Scientific), 5 μl MgCl_2_ (2.5 mM), 5 μl dNTPs (0.2 mM of each), 2 μl of each primer (0.4 pM μl^-1^), and 1 Unit *Taq* DNA polymerase (TrueStart Hot Start, Thermo Scientific). The amplifications were carried out on a T-GRADIENT thermocycler (Biometra, Göttingen, Germany) with the following thermal programmes: 95°C (2 min) for initial denaturation followed by 40 cycles of denaturation at 95°C (30 s), annealing at 52.5°C (ITS, LSU), 54°C (*rpb1*), 55.5°C (β*-tub*), 58.5°C (*act*) (40 s), extension at 72°C for 100 s (ITS and LSU), 80 s (*ß-tub*), and 60 s (*rpb1* and *act*), and a final extension at 72°C (10 min). PCR products were purified using the DNA Clean & Concentrator^TM^-5 kit (Zymo Research Corp., Irvine, California, USA) according to the manufacturer’s instructions. The cycle sequencing products were run on an ABI 3730XL sequencing machine (Eurofins Genomics GmbH, Germany). Obtained sequences were assembled, edited and trimmed with Sequencher 5.4.1 (Gene Codes Corporation, Ann Arbor, Michigan, USA) and deposited in GenBank under accession numbers KY607531–KY607585 and KY684180–KY684193.

### Alignment and phylogenetic reconstruction

Newly generated and already published sequences were used in phylogenetic analyses (Table 1). The latter were selected according to BLASTn searches (http://blast.ncbi.nlm.nih.gov/Blast.cgi) [41] that used the former as queries. Representatives of Bionectriaceae and Nectriaceae were selected mainly following Hirooka et al. [42] and Jaklitsch and Voglmayr [43]. Several data sets based on various combinations of ITS, LSU, *rpb1*, *act*, *ß-tub* sequences were compiled, aligned and analysed separately. Sequences of the LSU or their combination with *rpb1* (S1 Fig) or *rpb1* and *act* (S2 Fig) were used for above genus level phylogenetic inferences. Species level phylogenetic inferences were based on all five generated loci.

**Table 1:** Isolates and accession numbers used in the phylogenetic analyses.

Species	Isolate number	Host / substrate	Locality	GenBank accession numbers					Reference
				*act*	ITS	LSU	*rpb1*	*tub*	
***Aschersonia placenta***	BCC 7957	scale insect	-	-	-	DQ518753	DQ522364	-	[55]
***Balansia henningsiana***	GAM 16112	*Panicum* sp.	Georgia	-	-	AY489715	AY489643	-	[38]
***Balansia pilulaeformis***	AEG 94–2	Poaceae		-	-	AF543788	DQ522365	-	[55]
***Bionectria byssicola***	CBS 914.97 = G.J.S. 95–131	Branches of *Alchornea*	Uganda	GQ505962	-	GQ506011	GQ506040	-	[42]
***Bionectria compactiuscula***	CBS 592.93 = G.J.S. 93–27	-	France	GQ505963	-	GQ506007	GQ506036	-	[42]
***Bionectria epichloe***	CBS 118752	-		-	-	DQ363259	-	-	[43]
***Bionectria grammicospora***	G.J.S. 85–218	-	Indonesia	-	-	AF193238	-	-	[56]
***Bionectria ochroleuca***	CBS 125111	Palm branch	Costa Rica	GQ505964	-	GQ506009	GQ506038	-	[42]
***Bionectria pityrodes***	ATCC 208842	On bark	Mauritius	-	-	AY489728	AY489658	-	[38]
***Bionectria setosa***	CBS 834.91	*Throphis racemosa*	Cuba	-	-	AF210670		-	[57]
***Bionectria vesiculosa***	HMAS 183151	Decaying leaves of a dicotyledonous plant	China	-	-	HM050302	-	-	[58]
***Bryocentria brongniartii***	M190	-	UK	-	-	EU940125	-	-	[59]
***Bryocentria metzgeriae***	M140	-	Germany	-	-	EU940106	-	-	[59]
***Calonectria morganii***	ATCC 11614	Crown canker on *multiflora roses*	-	-	-	U17409	-	-	[43]
***Chaetopsinectria chaetopsinae***	Voucher 83362	-	-	-	-	DQ119554	-	-	[43]
***Clonostachys pityrodes***	ATCC 208842 = G.J.S. 95–26	On bark	Mauritius	-	-	AY489728	AY489658	-	[38]
***Cosmospora coccinea***	CBS 114050 = A.R. 2741	*Inonotus nodulosus*	Germany	GQ505967	-	GQ505990	GQ506020	HM484589	[42, 60]
***Cosmospora episphaeria***	G.J.S. 88–29	-	-	-	-	AY015625	-	-	[43]
***Cosmospora magnusiana***	CBS 129430 = A.R. 4453	*Rhus typhina*	USA	JF832441	-	JF832680	JF832764	JF832839	[61]
***Cosmospora vilior***	CBS 126109 = G.J.S. 90–217	*Xylaria* sp.	Venezuela	GQ505965	-	GQ506010	GQ506039	JF832840	[42, 61]
***Cosmospora vilior***	G.J.S. 96–186	-	-	-	-	AY015626	-	-	[43]
***Cosmospora viliuscula***	CBS 455.96 = G.J.S. 96–6	*Xylaria* sp.	Puerto Rico	GQ505966	-	GQ506003	GQ506032	HM484876	[42, 62]
***Cosmospora wegeliniana***	CBS 128986 = G.J.S. 93–15	*Diatrypaceae*	France	GQ505968	-	GQ506006	GQ506035	HM484878	[42, 62]
***Dialonectria episphaeria***	G.J.S. 10–193	*Diatrype stigma*	USA	-	-	KC291771	KC291892	KC291932	[63]
***Emericellopsis glabra***	CBS 125295 = A.R. 3614	Soil	Mexico	GQ505969	-	GQ505993	GQ506023	HM484879	[42, 62]
***Emericellopsis maritima***	CBS 491.71 = AFTOL-ID 999	See water	Ukraine	-	-	FJ176861	-	-	[43]
***Gliocephalotrichum bulbilium***	ATCC 22228	Soil	Louisiana, USA	-	-	AY489732	AY489664	-	[38]
***Gliomastix masseei***	CBS 794.69	Dung of rabbit	Italy	-	-	HQ232060	-	-	[64]
***Gliomastix murorum***	CBS 119.67	Camarophyllus niveus	Netherlands	-	-	HQ232066	-	-	[64]
***Gliomastix polychroma***	CBS 181.27	*Hevea brasiliensis*, bark	Sumatra	-	-	HQ232091		-	[64]
***Gliomastix roseogrisea***	CBS 279.79	Unknown	Switzerland	-	-	HQ232122	-	-	[64]
***Gliomastix tumulicola***	K5916-10-3	Viscous substances on stone wall	Japan	-	-	AB540476	-	-	[65]
***Heleococcum aurantiacum***	CBS 201.35	Mushroom compost	Unknown	-	-	JX158441	-	-	[66]
***Heleococcum japonicum***	CBS 397.67 = ATCC 18157	Wood panel of *Abies firma*	Japan	-	-	U17429	-	-	[43]
***Hydropisphaera multiloculata***	CBS 340.77	Dead leaf of *Astelia*	New Zealand	-	-	EU289204	-	-	[67]
***Hydropisphaera multiseptata***	CBS 336.77	*Phormium tenax*, Leaf	New Zealand	-		EU289205	-	-	[67]
***Hydropisphaera peziza***	CBS 102038	On bark	Alabama	-	-	AY489730	AY489661	-	[38]
***Hydropisphaera sp*. *(fungicola)***	CBS 122304 = A.R. 4170	Decaying leaves of *Populus trichocarpa*	Idaho	GQ505970		GQ505995	GQ506025	HM484877	[42, 62]
***Hydropisphaera suffulta***	CBS 136679 = CLLMAR13069	Pyrenomycetes on *Piper dilatatum*	Martinique	-	-	KU237206	-	-	Direct submission
***Hypocrella nectrioides***	G.J.S 89–104	scale insect	-	-	-	DQ518772	-	-	[55]
***Ijuhya antillana***	CBS 122797 = CLL 7321	Dead inflorescence of *Heliconia caribaea*	Martinique	KY607565	KY607537	KY607552	KY607578	KY684186	This study
***Ijuhya chilensis***	CBS 102803	Dead leaf	Texas; USA	KY607566	KY607538	KY607553	KY607579	KY684187	This study
***Ijuhya corynospora***	CBS 342.77 = G.J.S. 74–135	*Phormium tenax*, dead leaf	New Zealand	KY607567	KY607539	KY607554	KY607580	KY684188	This study
***Ijuhya dentifera***	CBS 574.76 = G.J.S. 74–43	*Dacrydium cupressinum*, bark	New Zealand	KY607568	KY607540	KY607555	KY607581	KY684189	This study
***Ijuhya faveliana***	CBS 133850 = CLLGUY12049	Palm	French Guiana	KY607569	KY607541	KY607556	KY607582	KY684190	This study
***Ijuhya faveliana***	CLLG10007	*Astrocarium* sp., dead leaf	French Guiana	-	-	KX950705	-	-	[68]
***Ijuhya fournieri***	CLLG12002	*Astrocarium* sp., dead leaf	French Guiana	-	-	KR105614	-	-	[69]
***Ijuhya fournieri***	CBS 128283 = CLLG10113	*Astrocarium* sp., dead leaf	French Guiana	-	-	KP899118	-	-	[69]
***Ijuhya lilliputiana***	CLLG12015B(LIP)	Palm, dead leaves	French Guiana	-	-	KX950703	-	-	[68]
***Ijuhya oenanthicola***	CBS 129747 = CLL10046	*Oenanthe crocata*	France	KY607570	KY607542	KY607557	KY607583	KY684191	This study
***Ijuhya pachydisca***	CLLG12001B	Palm, dead leaves	French Guiana	-	-	KX950701	-	-	[68]
***Ijuhya pachydisca***	CLLG12001B	Palm, dead leaves	French Guiana	-	-	KX950704	-	-	[68]
***Ijuhya paraparilis***	MAFF241404/TUAh52	-	Japan	GQ505971	-	GQ506012	GQ506041	-	[42]
***Ijuhya paraparilis***	W8063/HMAS 183506	-	China	-	FJ969801	HM050303	-	FJ969803	[70]
***Ijuhya parilis***	CBS 136677 = CLL13022	*Genista* sp.	Spain	KY607571	KY607543	KY607558	KY607584	KY684192	This study
***Ijuhya peristomialis***	CBS 569.76 = G.J.S. 73–314	rachis of *Cyathea dealbata*	New Zealand	KY607572	KY607544	KY607559	KY607585	KY684193	This study
***Ijuhya tetraspora***	CBS 140721	*Humulus lupulus*	Belgium	-	-	KX950706	-	-	[68]
***Ijuhya vitellina***	72723 (36_1G)	*Heterodera filipjevi*, egg	Turkey	KY607561	KY607532	KY607546	KY607574	KY684181	This study
***Ijuhya vitellina***	72825 (37AD)	*Heterodera filipjevi*, egg	Turkey	KY607562	KY607533	KY607547	KY607575	KY684182	This study
***Ijuhya vitellina***	72934 (37T)	*Heterodera filipjevi*, egg	Turkey	-	KY607534	KY607548	-	KY684183	This study
***Ijuhya vitellina***	DSM 104494 (41E)	*Heterodera filipjevi*, egg	Turkey	KY607563	KY607535	KY607549	KY607576	KY684184	This study
***Ijuhya vitellina***	DSM 104495 (42DD)	*Heterodera filipjevi*, egg	Turkey	KY607564	KY607536	KY607550	KY607577	KY684185	This study
***Ijuhya vitellina***	12-42-1e	*H*. *filipjevi* egg, uncultured	Turkey	-	-	KY607551	-	-	This study
***Ijuhya vitellina***	72918 (YE3T)	*Heterodera filipjevi*, egg	Turkey	KY607560	KY607531	KY607545	KY607573	KY684180	This study
***Kallichroma glabrum***	JK5123	-	Australia	-	-	AF193233	-	-	[56]
***Lanatonectria flocculenta***	CBS 126441 = G.J.S. 01–66	Bark	Ecuador	JF832481	-	JF832713	-	JF832913	[61]
***Lasionectria mantuana***	CBS 114291 = A.R. 4029	Decortivated wood	Finland	-	HM484858	GQ505994	GQ506024	-	[42, 62]
***Lasionectria marigotensis***	CBS 131606 = CLLGUAD11002	*Cocos nucifera*, decayding leaf	French West Indies	-	KR105612	KR105613	-	-	[71]
***Lasionectria sylvana***	CBS 566.76	*Cyathea smithii*	New Zealand	-	-	EU289206	-	-	[67]
***Moelleriella libera***	P.C.672 = CUP 067773	-	Honduras	-	-	EU392594	EU392717	-	[72]
***Mycoarachis inversa***	ATCC 22107 = A.R. 2745	Elephant dung	Uganda	GQ505972	-	GQ505991	GQ506021	HM484882	[42, 62]
***Nectria aurantiaca***	CBS 308.34	*Ulmus* sp.	UK	JF832482	-	JF832682	JF832766	JF832886	[61]
***Nectria austroamericana***	CBS 126114 = A.R. 2808	*Gleditsia triacanthos*	USA	GQ505960	-	GQ505988	GQ506016	HM484597	[42, 62]
***Nectria balansae***	CBS 123351 = A.R. 4446	*Coronilla* sp.	France	GQ505977	-	GQ505996	GQ506026	HM484607	[42, 60]
***Nectria berberidicola***	CBS 128669 = A.R. 4662	*Berberis vulgaris*	France	JF832487	-	JF832712	JF832767	JF832887	[61]
***Nectria berolinensis***	CBS 127382	*Ribes sanguinea*	Austria	-	-	HM534893	-	-	[73]
***Nectria cinnabarina***	CBS 255.47 = ATCC 11432	Stem of *Ulmus* sp.	Netherlands	GQ505975	-	GQ505997	GQ506027	HM484832	[42, 60]
***Nectria cucurbitula***	CBS 259.58	*Pinus sylvestris*	Netherlands	GQ505974	-	GQ505998	GQ506028	HM484592	[42, 62]
***Nectria cyanostoma***	CBS 101734 = G.J.S. 98–127	*Buxus sempervirens*	France	GQ505961	-	FJ474081	GQ506017	HM484611	[42, 62]
***Nectria haematococca***	CBS 114067	On bark	Guyana	-	-	AY489729	AY489660	-	[38]
***Nectria lamyi***	CBS 115034 = A.R. 2779	*Berberis vulgaris*	Austria	HM484507	-	HM484569	HM484582	HM484593	[60]
***Nectria pseudotrichia***	CBS 551.84	Bark	Japan	GQ505976	-	GQ506000	GQ506030	HM484602	[60]
***Nectria pseudotrichia***	CBS 641.83	Wood	Venezuela	-	-	HM534899	-	-	[43]
***Nectria sinopica***	CBS 462.83	*Hedera helix*	Netherlands	GQ505973	-	GQ506001	GQ506031	HM484595	[60]
***Nectricladiella camelliae***	CBS 111794 = ATCC 38571	Fruit of tree	Australia	-	-	AY793432	-	-	[43]
***Nectriopsis epimycota***	CBS 127459 = G.J.S. 95–94	Pyrenomycete	Puerto Rico	GQ505978	-	GQ506008	GQ506037	-	[42]
***Nectriopsis exigua***	CBS 126110 = G.J.S. 98–32	Myxomycete	Puerto Rico	GQ505979	-	GQ505986	GQ506014	HM484883	[42, 62]
***Nectriopsis violacea***	CBS 424.64	*Fuligo septica*	Germany	-	-	AY489719	AY489646	-	[38]
***Neocosmospora vasinfecta***	JP963	-	-	-	-	U17406	-	-	[43]
***Neonectria coccinea***	CBS 119158 = G.J.S. 98–114	*Fagus* sp.	Germany	KC660422	-	KC660620	KC660672	KC660727	[74]
***Neonectria ditissima***	CBS 100316	*Malus domestica*	Ireland	HM352880	-	HM364311	HM364330	HM352864	[62]
***Neonectria punicea***	MAFF241548 = TPP-h328	Twigs	Japan	KC660372	-	KC660569	KC660637	KC660713	[74]
***Neonectria veuillotiana***	CBS 125114 = G.J.S.92-24	*Fagus sylvatica*	France	GQ505980	-	GQ506005	GQ506034	JQ394725	[42, 75]
***Neonectria westlandica***	CBS 112464 = G.J.S. 83–156	*Dacrydium cupressinum*	New Zealand	GQ505959	-	GQ505987	GQ506015	HM484610	[60]
***Nigrosabulum globosum***	ATCC 22102	Cow dung	USA: Wyoming	-	-	AF096195	-	-	[43]
***Ochronectria calami***	CBS 125.87	On palm	Indonesia	-	-	AY489717	AY489644	-	[38]
***Ochronectria thailandica***	MFLUCC 15–0140	-	Thailand	-	-	KU564069	-	-	Direct submission
***Ophionectria trichospora***	CBS 109876 = G.J.S. 01–206	Bark	Cameroon	-	-	AF543790	AY489669	HM484886	[62]
***Paracylindrocarpon aloicola***	CBS 141300 = CPC 27362	Leaves and twigs *of Aloe* sp.	South Africa	-	-	KX228328	-	-	[76]
***Persiciospora africana***	ATCC64691	Forest soil	Botswana	-	-	AY015631	-	-	[43]
***Pleonectria aquifolii***	CBS 307.34	*Ilex aquifolium*	UK	JF832444	-	JF832718	JF832792	JF832842	[61]
***Pleonectria coryli***	CBS 129358 = A.R. 4583	*Corylus avellana*	France	JF832476	-	JF832740	JF832797	JF832872	[61]
***Protocreopsis korfii***	CBS138733 = CLLM14077	-	-			KT852955			Direct submission
***Protocreopsis pertusa***	C.T.R. 72–184	Decaying palm	Venezuela	GQ505981	-	GQ506002	-	-	[42]
***Pseudonectria pachysandricola***	CBS 128674 = A.R. 4592	*Pachysandra* sp.	USA	JF832512	-	JF832715	JF832791	JF832909	[61]
***Pseudonectria rousseliana***	CBS 114049	*Buxus sempervirens*, leaf	Spain	-	-	U17416	AY489670	-	[38]
***Roumegueriella rufula***	G.J.S.91-164	*Globodera rostochiensis*	-	-	-	EF469082	EF469099	-	[77]
***Roumegueriella rufula***	CBS 346.85	*Globodera rostochiensis*	Switzerland	-	-	GQ505999	GQ506029	-	[42]
***Selinia pulchra***	A.R. 2812	Cow dung	Argentina	GQ505982	-	GQ505992	GQ506022	HM484884	[42, 62]
***Stephanonectria keithii***	CBS 114057	*Eleagnus* sp., bark	France	-	-	AY489727	AY489657	-	[38]
***Stilbocrea macrostoma***	CBS 114375 = G.J.S. 73–26	*Geniostoma ligustifolia*	New Zealand	-	-	AY489725	AY489655	-	[38]
***Stilbocrea macrostoma***	G.J.S. 02–125	-	Sri Lanka	GQ505983	-	GQ506004	GQ506033	-	[42]
***Stromatonectria caraganae***	CBS 125579	Branches of *Caragana* spp.	Austria	-	HQ112288	HQ112288	-	HQ112289	[43]
***Stromatonectria caraganae***	CBS 127387	Branches of *Caragana* spp.	Austria	-	HQ112287	HQ112287	-	-	[43]
***Verrucostoma freycinetiae***	MAFF240100/h523	*Freycinetia boninensis*	Japan	GQ505984	-	GQ506013	GQ506018	HM484885	[42, 62]
***Viridispora alata***	CBS 125123 = A.R. 1770	Bark	Madeira	GQ505985	-	GQ505989	GQ506019	JF832912	[61]
***Viridispora diparietispora***	CBS 102797 = ATCCMYA627	*Crataegus crus-galli*	USA	-	-	AY489735	AY489668	-	[38]

**AEG**: A. E. Glenn personal collection; **ATCC**: American Type Culture Collection, Manassas, VA, USA; **A.R.**: Amy Y. Rossman, USDA-ARS MD USA; **BCC**: BIOTEC Culture Collection, Pathum Thani, Thailand; **CBS**: Westerdijk Fungal Biodiversity Institute (Utrecht, The Netherlands); **C.L.L.**:Christian Lechat, Ascofrance, Villiers en Bois, France.; **C.T.R.**: Clark T. Rogerson, The New York Botanical Garden, NY, USA; **DSM**: The open collection of the Leibniz-Institut DSMZ- Deutsche Sammlung von Mikroorganismen und Zellkulturen GmbH; **GAM**: Julian H. Miller Mycological Herbarium Athens, GA, USA; **G.J.S.**: Gary J. Samuels, USDA-ARS MD USA; **MAFF**: MAFF Genebank, National Institute of Agrobiological Sciences, Ibaraki, Japan; **HMAS**: The mycological Herbarium, Institute of Microbiology, Chinese Academy of Sciences, China; **PC**: Herbier Cryptogamique, Départment de Systématique et Évolution, Muséum National d’Histoire Naturelle, Paris, France.

DNA sequences were aligned using the online version of Mafft v.7 [44] adopting the iterative refinement algorithms L-INS-I for *rpb1*-, *act*-, and *ß-tub* gene regions and Q-INS-i for LSU and ITS. The start and end of the alignments were trimmed manually in Se-Al v2.0 [45]. The alignments were deposited in TreeBASE, and are available at (http://purl.org/phylo/treebase/phylows/study/TB2:S20879). Based on these alignments, Bayesian Metropolis coupled Markov chain Monte Carlo analyses were done with MrBayes v3.2 [46, 47]. The general time-reversible model with the addition of invariant sites and a gamma distribution of rates across sites (GTR+I+G) was selected as the best fitting substitution model according to both the hierarchical likelihood ratio test (hLRT) and the Akaike Information Criterion (AIC) implemented in MrModeltest v2.2 [48]. Starting with a randomly selected tree, 1.000.000 (for the two- and five-gene-data set) 2.000.000 (for the three-gene data set) and 5.000.000 generations (for the LSU data set) were run, using flat *prior* distributions. Trees were sampled every 500 generations and 50% majority rule consensus trees were computed and *a postieriori* probabilities (pp) were estimated only from trees of the plateau, and after the split frequencies had fallen below 0.01. All other trees were discarded as “burnin”. The estimations were thus based on 1600 (two- and five-gene data set), 2300 (three-gene data set) and 7000 (LSU) trees sampled. Maximum likelihood (ML) analyses were performed using RAxML 7.2.8 [49, 50] implemented in Geneious 8.1.2 applying the general time-reversible (GTR) substitution model with gamma model of rate heterogeneity and 100 replicates of rapid bootstrapping (reported as MLB values). Neighbor joining analysis [51] was done in PAUP 4.0b10 in the batch file mode [52] applying the Kimura two-parameter model of DNA substitution [53] with a transition/transversion ratio of 2.0 to compute genetic distances. Support for internal nodes was estimated by 1000 bootstrap replicates [54] (reported as NJB values). The phylogenetic trees were visualised using FigTree v. 1.4.2 (http://tree.bio.ed.ac.uk/software/figtree).

### Nomenclature

The electronic version of this article in Portable Document Format (PDF) in a work with an ISSN or ISBN will represent a published work according to the International Code of Nomenclature for algae, fungi, and plants, and hence the new names contained in the electronic publication of a PLOS article are effectively published under that Code from the electronic edition alone, so there is no longer any need to provide printed copies. In addition, new names contained in this work have been submitted to MycoBank from where they will be made available to the Global Names Index. The unique MycoBank number can be resolved and the associated information viewed through any standard web browser by appending the MycoBank number contained in this publication to the prefix http://www.mycobank.org/MB/. The online version of this work is archived and available from the following digital repositories: PubMed Central, LOCKSS.

### Metabolite profiling

#### Fermentation and extraction of cultures

Three different liquid media (Q6/2, YM and ZM; [78]) were used for the initial screening of fermentation in 500 mL Erlenmeyer flasks each containing 200 mL medium. Inoculum consisted of few 5-mm-diam. culture discs of strain DSM 104495 excised from PDA. The submerged cultures were incubated in the dark at 23°C and 140 rpm and harvested two days after sugars were depleted. Secondary metabolites were extracted from both mycelium and culture filtrate following methods described by Kuhnert et al. [78]. For large-scale fermentation, the above mentioned strain was inoculated into 3 L of the selected medium (see below) and processed for extraction similarly.

#### Selection of the liquid culture for scale-up culturing

Minimum inhibitory concentration (MIC) tests were performed to determine the optimum medium with the highest antimicrobial activity in crude extracts. The cultural medium showing highest MIC activity was chosen for scale-up fermentation, accordingly. The crude extracts obtained from the examined cultural media *i*.*e*. Q6/2, YM and ZM were tested following Chepkirui et al. [79].

#### Isolation of secondary metabolites

The EtOAc organic extract (110 mg) was dissolved in MeOH and purified using preparative RP-HPLC [column 250 × 20 mm, Kromasil C_18_, 7 μm; equipped with a Kromasil C_18_ pre-column 50 x 20 mm, 7 μm]. Solvent A: H_2_O; solvent B: acetonitrile; gradient: 50% B increasing to 80% B in 40 min, increasing to 100% B in 5 min, holding at 100% B for 10 min; flow rate 20 mL/min, UV detection at 230, 254, and 325 nm], yielded 2.3 mg of compound **1** and 2 mg of compound **2**. The compounds were eluted at 32 min and 43 min, respectively.

#### Structure elucidation

1D and 2D NMR spectra were recorded on a Bruker Avance III 700 spectrometer with a 5 mm TXI cryoprobe (^1^H 700 MHz, ^13^C 175 MHz) spectrometer; optical rotations were measured on a Perkin-Elmer 241 polarimeter. All HPLC-MS analyses were performed on Agilent 1260 Infinity Systems with diode array detector and C_18_ Acquity UPLC BEH column (2.1 x 50 mm, 1.7 μm) from Waters with the gradient described by Helaly et al. [80], combined with ion trap MS (amazon speed, Bruker); and HR-ESIMS spectra on a time-of-flight (TOF) MS (Maxis, Bruker). Chemicals and solvents were obtained from AppliChem GmbH, Avantor Performance Materials, Carl Roth GmbH & Co. KG and Merck KGaA in analytical and HPLC grade.

#### Nematode bioassays and cytotoxicity

A biological assay was conducted to evaluate nematicidal activity of pure compounds against *Caenorhabditis elegans* and *H*. *filipjevi* according to Helaly et al. [80]. Surface-sterilised cysts of *H*. *filipjevi* propagated in the greenhouse were incubated in sterile tap water under aseptic conditions for nematode hatching. The freshly hatched second stage juveniles (J2) were collected and used for the experiment. *Caenorhabditis elegans* was cultivated as described by Helaly et al. [80]. The number of nematodes was adjusted to 600/mL of J2 of *H*. *filipjevi* and 600/mL of adults and juveniles of *C*. *elegans* in sterile tap water. The assays were carried out in 24-well microtiter plates. Each well received 1 mL of nematode suspension. The compounds were tested against nematodes at the concentrations of 100, 50, 20 and 10 μg/mL in MeOH. Each treatment included three replications. Ivermectin (Sigma-Aldrich) was used as positive and MeOH in DMSO (v/v) as negative control. Nematodes were monitored for 30 min after inoculation and afterwards plates were incubated at 24°C for 18 h. Cytotoxicity (IC_50_) of compounds was tested against different cell lines as described by Richter et al. [81].

## Results

### Fungal isolation

**Fig 1:**
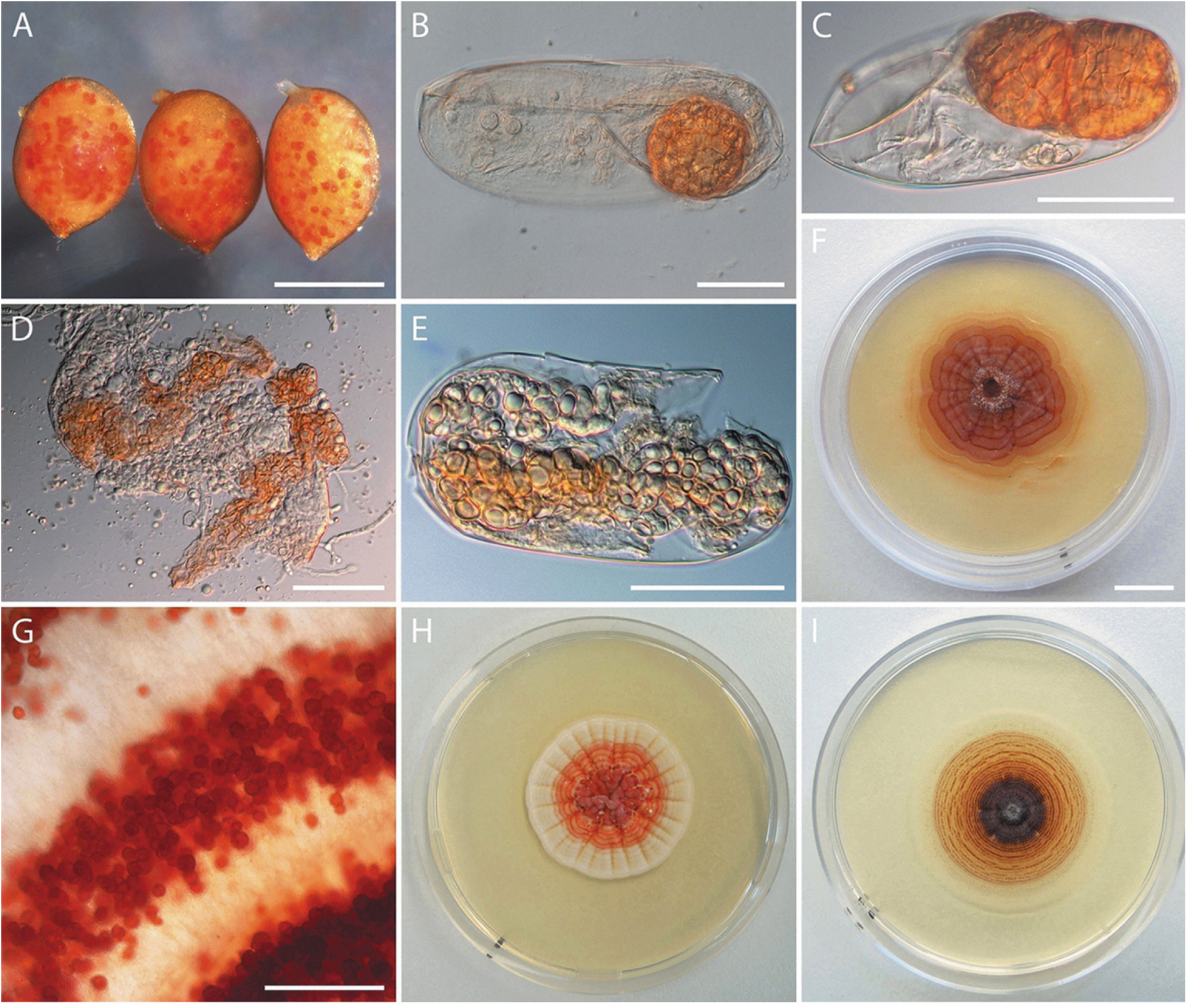
Cysts and eggs of *Heterodera filipjevi* naturally infected with *Ijuhya vitellina*, and pure cultures obtained from the infected eggs. (A) Symptomatic, reddish dotted nematode cysts. (B, C) Nematode eggs accommodating reddish microsclerotia. (D, E) Microsclerotial tissue developing inside juveniles. (F) A six-month-old culture that developed from a single infected nematode egg. (G) Surface of colony showing reddish microsclerotia arranged in concentric rings. (H, I) Two-month-old cultures on PDA and CMA. Scale bars: A = 0.5 mm, B = 30 μm, C-E = 50 μm, F = 1 cm (also applying for H, I), G = 400 μm.

Among other nematode egg colonizing fungi to be described elsewhere, *Ijuhya vitellina*, newly described below, was encountered. It rendered nematode cysts collected from fields in Turkey reddish dotted upon microscopic examination (Fig 1A). Reddish dots inside cysts consisted of nematode eggs each containing one or few microsclerotia (Fig 1B and 1C). In some infected eggs, microsclerotial tissues were found developing inside juveniles (Fig 1D and 1E). When inoculated on agar medium (PDA), mycelium emerged from symptomatic nematode eggs and developed reddish orange, brick red or reddish brown cultures (Fig 1F). Predominantly globose or ellipsoidal, reddish microsclerotia were formed on the surface of (Fig 1G) or submerged in the medium. Microsclerotia formed *in vitro* resembled the structures encountered in nematode eggs. Cultures growing from individually inoculated nematode eggs or *in vitro-*produced microsclerotia developed slowly and reached a diameter of 2.8 cm within 3 months (Fig 1F, 1H and 1I). The sterility check revealed no fungal growth from the examined cysts.

### Molecular phylogenetic studies

#### DNA sequence comparisons and culture-independent identification

LSU sequences were obtained for all 14 studied strains of the fungus. Four of these LSU sequences were obtained from environmental specimens (individual nematode eggs showing reddish microsclerotia) were identical to those retrieved from pure cultures. One (2, 2, 3) nucleotide substitutions were observed among retrieved *act* (ITS, *ß-tub*, *rbp1*) sequences. Most substitutions were observed in strain 37AD.

BLASTn searches indicated relatedness of the encountered fungus to the Bionectriaceae. More specifically, the LSU sequence of *I*. *vitellina* was most similar to that of *Ijuhya paraparilis* and its ITS sequence to that of *Stromatonectria caraganae* according to initial searches in GenBank.

#### Phylogenetic reconstructions

Alignment of 112 LSU sequences representing 100 taxa of Nectriaceae and Bionectriaceae was 860 sites long. That of 66 combined LSU (844 sites) and *rpb1* (682 sites) sequences comprised 60 taxa, and that of 70 combined LSU (848), *act* (607) and *rpb1* (684) sequences represented 64 taxa. One alignment of 15 strains representing 10 species of the genus *Ijuhya* only was based on all five gene regions sampled: *act* (624), ITS (936), LSU (819), *rpb1* (712) *ß-tub* (875).

**Fig 2:**
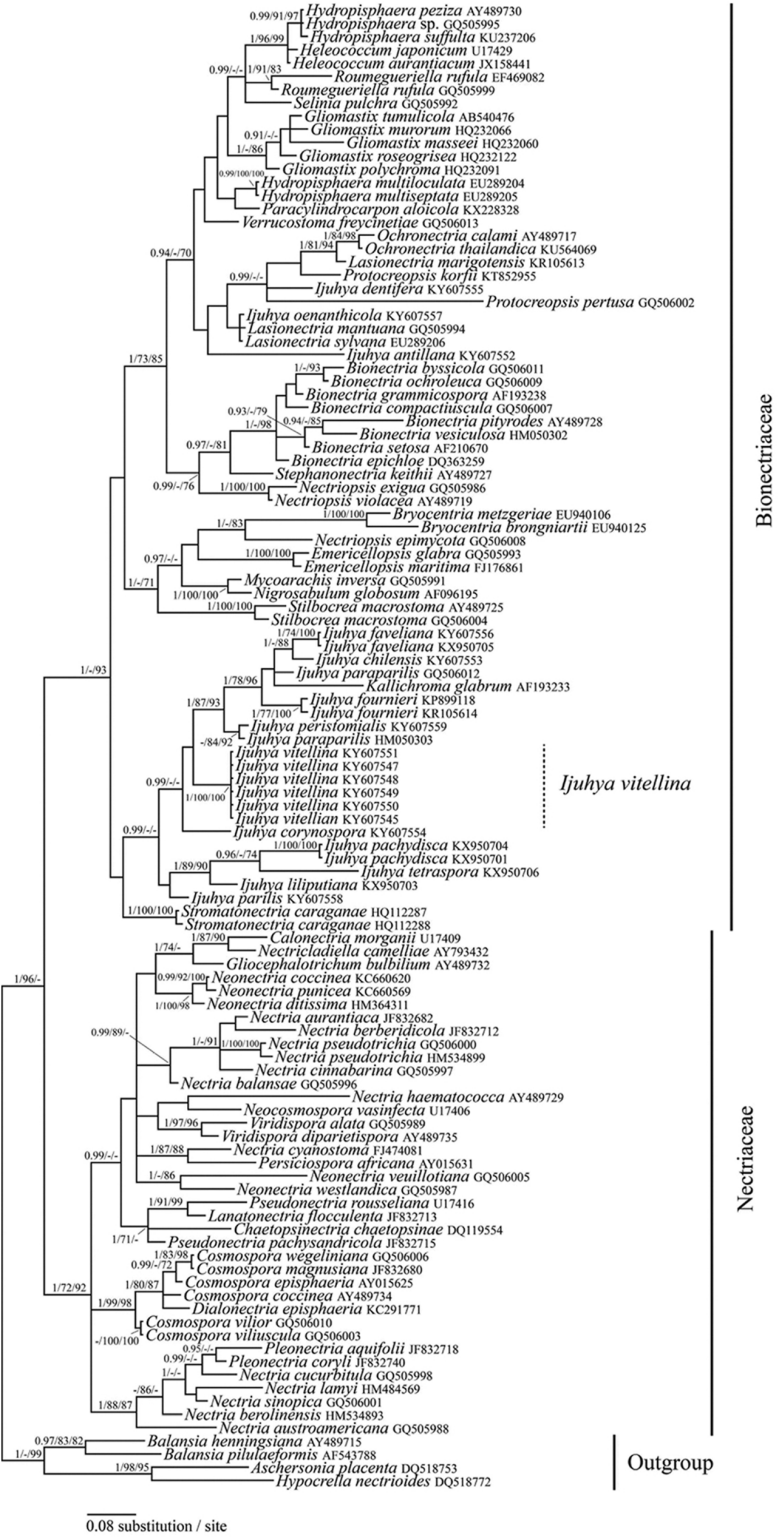
Bayesian inference of phylogenetic relationships of selected taxa of the Bionectriaceae and Nectriaceae (Hypocreales) based on LSU sequences. Numbers above nodes are estimates of *a posteriori* probabilities (≥ 0.9) / NJB and MLB values (≥70%). The topology was rooted with *Aschersonia placenta*, *Balansia henningsiana*, *B*. *pilulaeformis*, and *Hypocrella nectrioides*, (Hypocreales).

Strains of the nematode parasite were highly supported as a monophyletic species group in all analyses, and clustered within a highly supported clade together with other species of the bionectriaceous genus *Ijuhya* (Fig 2 and S1 and S2 Figs) including *I*. *peristomialis*, a later synonym of *I*. *vitrea*, which is the type species of *Ijuhya* [82]. Relatedness of *Ijuhya* with selected taxa of the Bionectriaceae, including Bionectria, the type genus of this family and various others was also highly supported. Phylogenetic analyses based on LSU only, LSU/*rpb1* and LSU/*rpb1*/*act* suggested that *Ijuhya oenanthicola*, *I*. *dentifera* and *I*. *antillana* are distantly related to *Ijuhya sensu stricto*. Instead, they display closer phylogenetic affinities to *Lasionectra* and *Ochronectria* (Fig 2 and S1 and S2 Figs).

**Fig 3:**
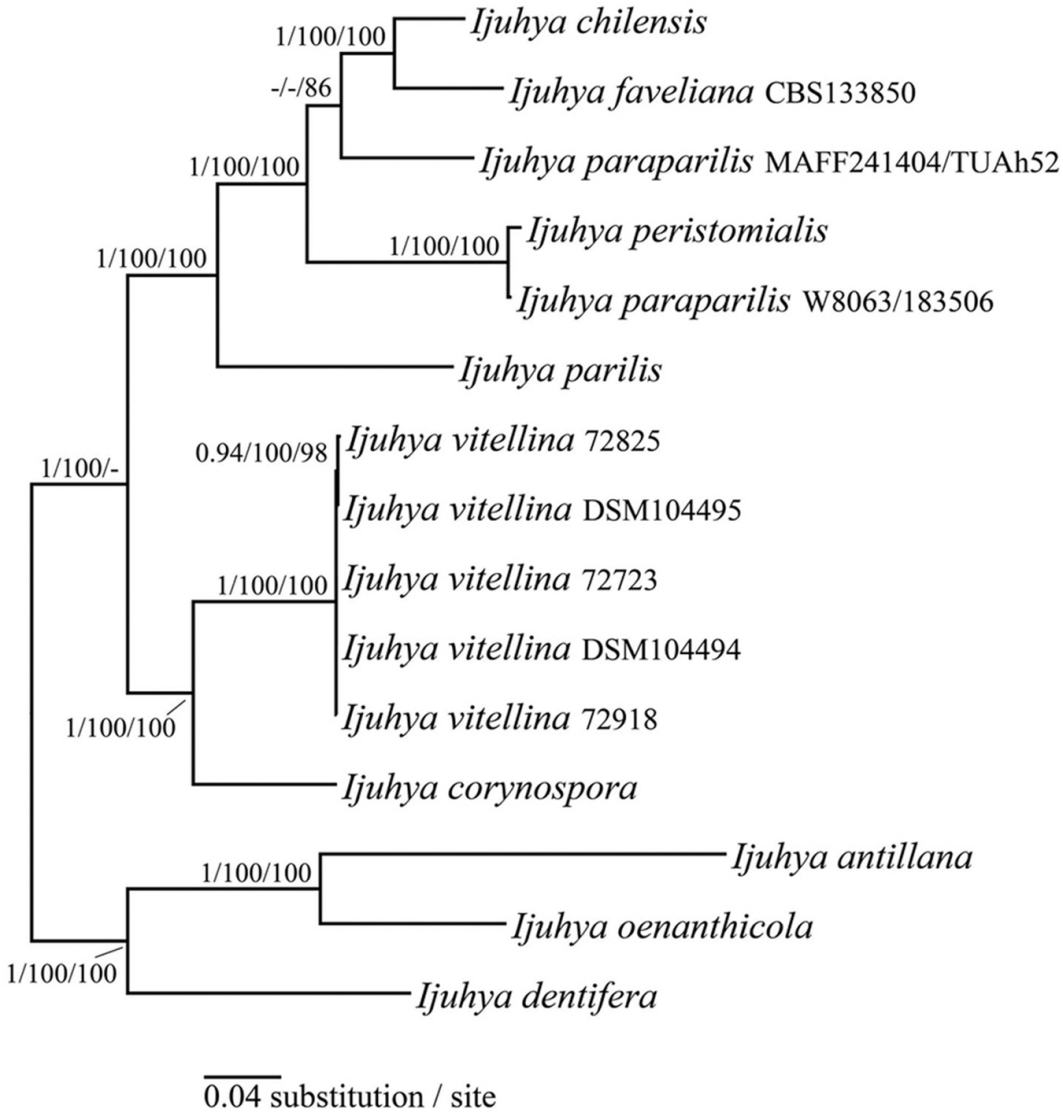
Bayesian inference of infrageneric phylogenetic relationships within *Ijuhya* based on *act*, ITS, LSU, *rpb1*, and *ß-tub* sequences. Numbers above nodes are estimates of *a posteriori* probabilities (≥ 0.9) / NJB and MLB (≥ 70%). The topology was rooted with three distantly related ‘*Ijuhya*’ species (‘*Ijuhya*’ *antillana*, *I*. *dentifera*, and ‘*Ijuhya*’ *oenanthicola*).

All five loci were sampled for the *Ijuhya* species available as cultures to us. Hypotheses on the intra-generic phylogenetic relationships of representatives of *Ijuhya* were derived using these sequences. In addition two specimens of *I*. *paraparilis* from GenBank, for which three of these five gene regions were available, were added to this data set. Based on the results of the larger phylogenies, the ‘*Ijuhya’* species distantly related to the *Ijuhya sensu stricto* were used as outgroup here. Two highly supported subclades are suggested for the in-group of genus *Ijuhya*, of which one includes the type species *I*. *peristomialis*, and in addition *I*. *chilensis*, *I*. *faveliana*, *I*. *paraparilis*, and *I*. *parilis* (Fig 3). The other subclade includes *I*. *vitellina* and its closest sister species, *I*. *corynospora* (Fig 3). This is in concordance with the phylogenetic hypotheses obtained from the larger two- and three-gene data sets (S1 and S2 Figs). The two isolates of *I*. *paraparilis* originating from Japan and China, did not form a species clade and are thus unlikely conspecific.

### Taxonomy

*Ijuhya vitellina* Ashrafi, W. Maier & Schroers, sp. nov., Mycobank MB 821493

Holotype for *Ijuhya vitellina* (here designated) (MB 821493): Turkey, Yozgat, experimental wheat field: dried culture on SNA with carnation leaf pieces, originating from an individual egg from a cyst of *Heterodera filipjevi*, isolated by *Samad Ashrafi*, August 2013 (B 70 0016479, deposited at the herbarium of the Botanic Garden and Botanical Museum Berlin-Dahlem).

Ex-holotype strain: DSM 104494, deposited in the open collection of the Leibniz-Institut DSMZ- Deutsche Sammlung von Mikroorganismen und Zellkulturen GmbH). Ex-holotype sequences: *act*: KY607563; ITS: KY607535; LSU: KY607549; *rpb1*: KY607576; *tub*2: KY684184.

Additional material examined, from the same location: DSM 104495 (dried culture, B 70 0016480), GenBank accession number: *act*: KY607564; ITS: KY607536; LSU: KY607550; *rpb1*: KY607577; *tub*2: KY684185. For other material studied, see Table 1.

**Etymology**: From Latin *vitellus* meaning egg yolk, referring to the colour and shape of microsclerotia formed by the species in nematode eggs.

Naturally infected eggs typically accommodating one, occasionally two globose to subglobose, fulvous (brownish-yellow, reddish yellow, yolk-coloured) multicellular microsclerotia with a *textura angularis* appearance, similar to microsclerotia formed in culture.

**Fig 4:**
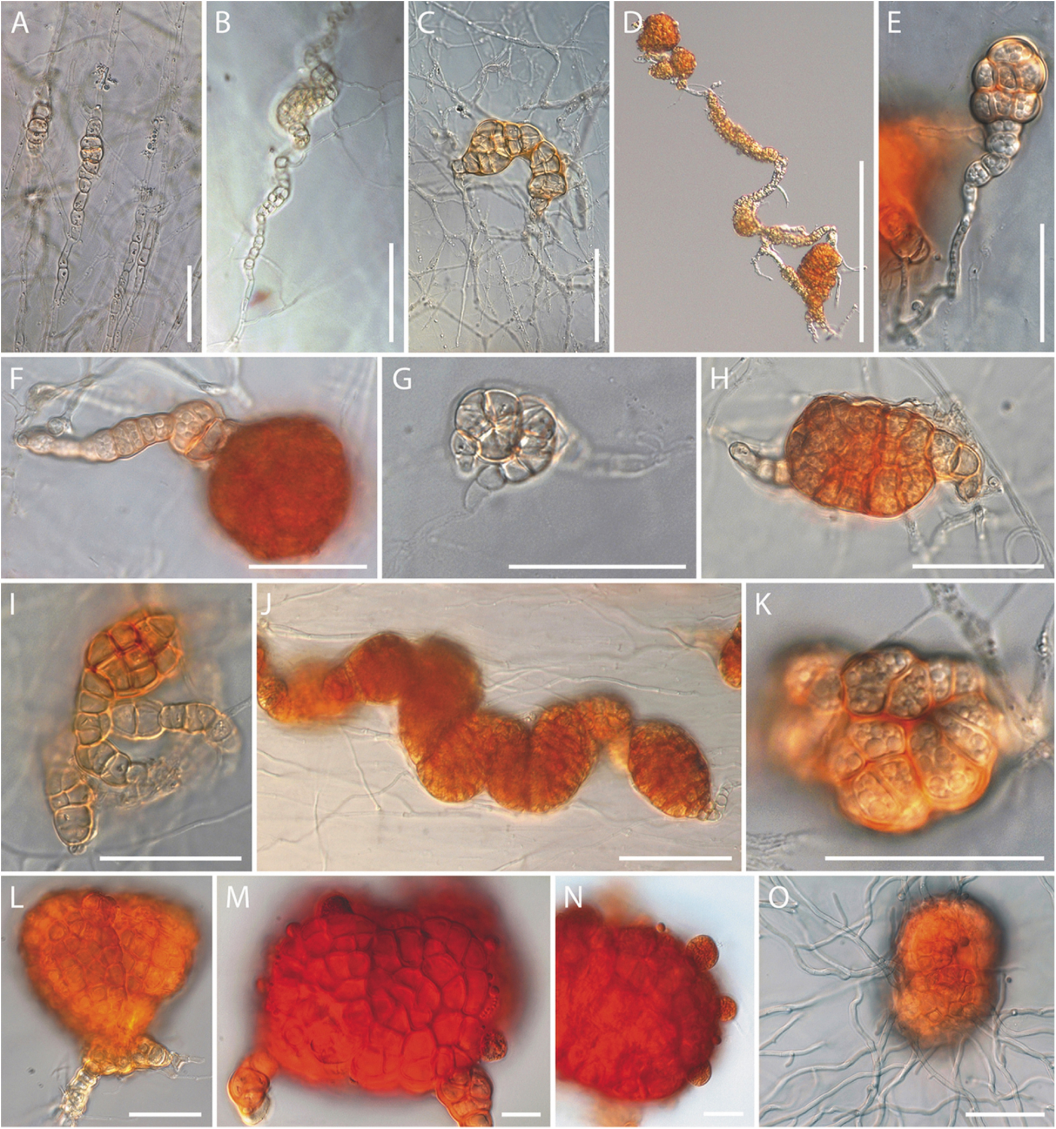
Light micrographs of *Ijuhya vitellina*, formation of microsclerotia. (A-F) Transformation of hyphae into (A-D) chlamydospore or dictyochlamydospore-like structures, and (E, F) microsclerotia. (G-I) Coiling or coalescence of dictyochlamydospore-like structures. (J) Microsclerotia densely arranged in a chain. (K-N) Pigmentation first observed (K) in cell walls, and later (L-M) intensifying throughout microsclerotia. (O) A single microsclerotium inoculated on agar surface developing hyphae. A-I, K-N: from PDA, J: from CMA, O: from PDA 1/3. Scale bars: (A, C, E-I, K, L, O) = 30 μm; B, J = 50 μm; D = 200 μm; (M, N) = 10 μm.

**Fig 5:**
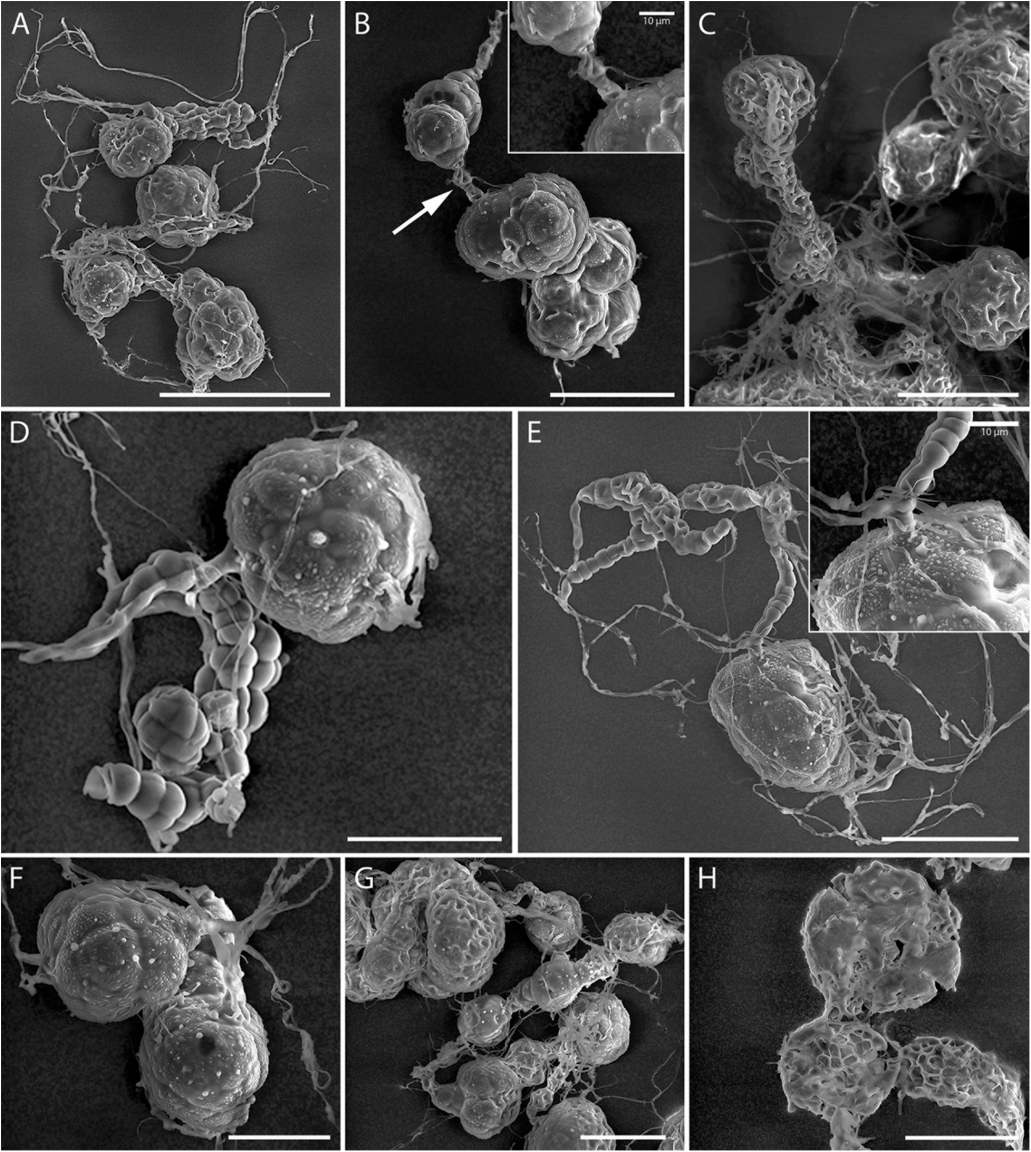
SEM micrographs of microsclerotia formed by *Ijuhya vitellina*. (A) Filamentous hyphae developing into multicellular structures. (B) Intercalary formed dictyochlamydospores connected by hyphae (arrowed). (C) Detail of intercalary multicellular structures of microsclerotia. (D, E) Hyphae transformed into chlamydospore-like structures and microsclerotia. (F) Terminally formed microsclerotium. (G) Moniliform arrangement of microsclerotia. (H) Detail of microsclerotia illustrating a multicellular surface that forms a *textura angularis*. Scale bars: A = 100 μm; (B, E-H) = 50 μm; (C, D) = 30 μm.

Colonies slow in growth; at 20°C on PDA, 6.5–9 mm diam. after 7 d, 13–16 mm after 14 d; optimum temperature for growth around 25°C, 9–12 mm (7 d), 16–19 mm (14 d); at 30°C 7–8 mm (7 d), 10–13 mm (14 d); no growth observed at 35°C. Colony reverse on OA after 21 d reddish orange (7A7), brick red to burnt Sienna (7B–D7–8) to dark brown (7F7–8) in central parts of colonies; on CMA and SNA covered with carnation leaf pieces Sahara (6C5), brick red or burnt Sienna (7B–D7–8); on up to 12 months old PDA caramel brown to brownish orange (6C6–8), cognac brown (6E7), brownish orange, brick red, copper red (7C–E7–8), or dark brown (7F6). Colony surface of similar pigmentation as reverse, granular because of solitary, gregarious or clustered microsclerotia formed on the surface of or submerged in the agar media, often arranged in concentric rings. Aerial mycelium on PDA within 3 wk not observed or sparsely to abundantly produced in sectors, white, felty to wet-cottony, on SNA and SNA with carnation leaf pieces absent or present as occasionally formed solitary, erect, typically apically coiling hyphae. Conidiophores and conidia not observed. Microsclerotia typically ellipsoidal to cylindrically oblong, sometimes globose, orange to brownish orange or brick red, not changing colour in KOH, on 6 wk-old OA 23–51 × 26–66 (36 × 44) μm (n = 52), on 6 wk-old PDA 25–46 × 32–58 (35 × 43) μm (n = 32), on ca. 12 months-old PDA cultures 26–58 × 34–77 (42 × 52) μm (n = 63). Cells of microsclerotia angular, forming a *textura angularis* in surface and subsurface view, variable in size, on 21 d old OA colonies 3.5–7.0 × 5.0–9.0 (5.0 × 7.0) (n = 41), on 21 d old PDA colonies 3.1–6.0 × 4.0–8.0 (5.0 × 6.0) μm (n = 50), on 12 month-old PDA colonies 3.0–7.0 × 4.5–11.0 (5.0 × 7.0) μm (n = 74) μm; walls first hyaline, later orange to brownish orange, 1.0–2.0 μm (n = 70). Microsclerotia developing from intercalary cells of hyphae or terminally at side branches, solitary or moniliform, first chlamydospore or dictyochlamydospore-like (Fig 4A–4D and Fig 5A–5C), cells angular, pigmented, first pale luteous, then brick reddish (Fig 4K–4N), filled with small guttules. Dictyochlamydospore-like structures may enlarge through coiling or expand to form globose to ellipsoidal microsclerotia (Fig 4E and 4F and Fig 5D–5F) formed solitary, in chains (Fig 4J and Fig 5G), or clusters. Globose and ellipsoidal microsclerotia developing in culture reminiscing microsclerotial structures of the fungus encountered in field-collected nematode eggs. Culturing of single microsclerotia directly extracted from nematode eggs, or retrieved from one-year old wheat straw cultures resulted in fungal growth (Fig 4O). Teleomorph not observed.

### *In vitro* parasitism on nematode eggs and Koch’s postulates

**Fig 6:**
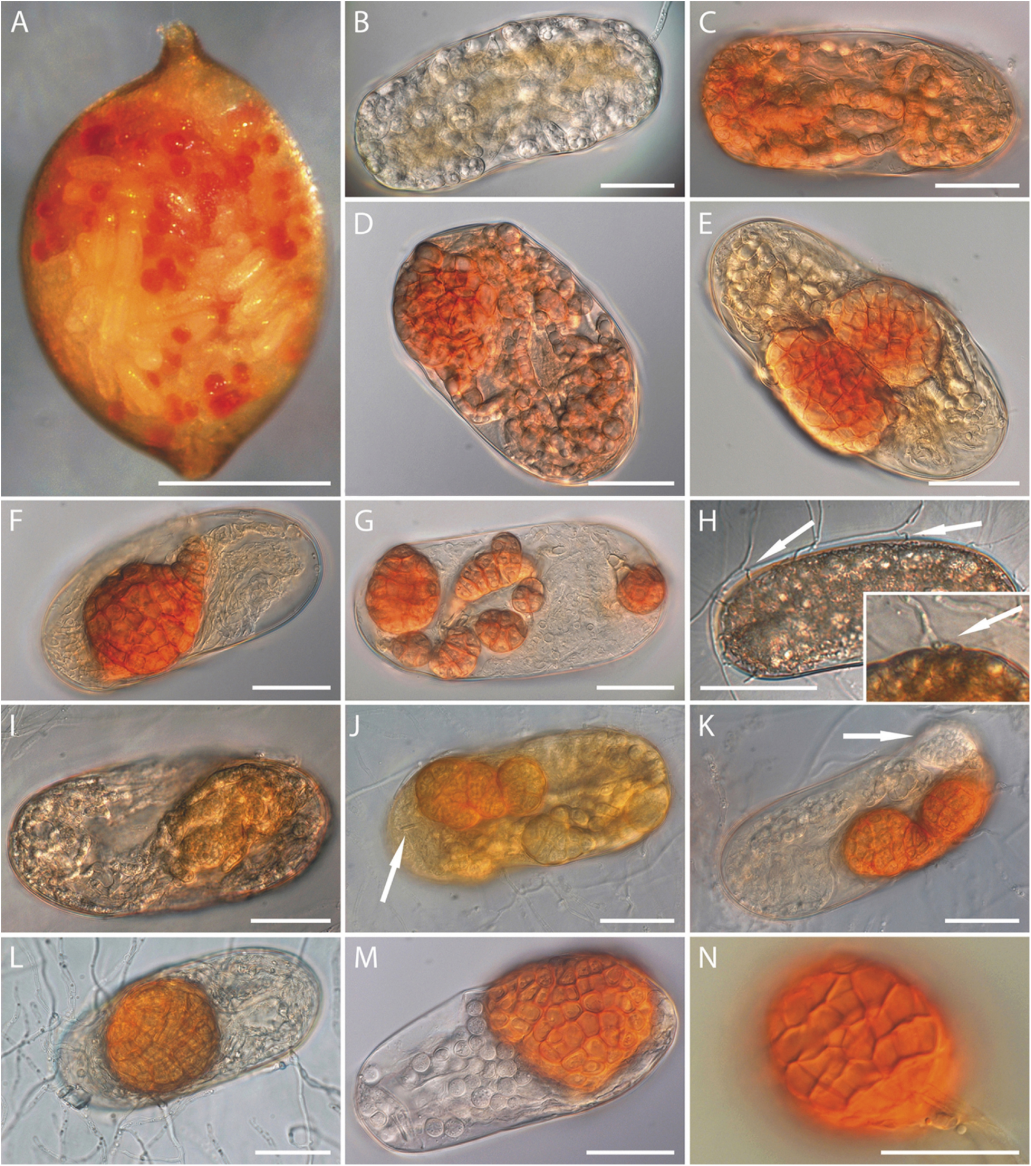
Light micrographs of the infection and colonisation process of *Ijuhya vitellina* in cysts and eggs of *Heterodera filipjevi*. (A) Symptomatic cyst, reddish-dotted due to eggs containing reddish, globose microsclerotia. (B-E) Early colonisation of nematode eggs by hyphae becoming chlamydospore- and dictychlamydospore-like to develop microsclerotia inside eggs. (F, G) Dictyochlamydospore-like structures and small microsclerotia. (H) Hyphae penetrating through the eggshell by forming appressorium-like structure (arrows). (I-K) Development of the fungus inside nematodes eggs: (I) Formation of thick-walled hyphal cells, later (J-K) transforming into microsclerotia. The arrow in (J) points at the nematode stylet; in (K) at immature microsclerotium. (L) Egg with mature microsclerotium. (M-N) Near-identical cells of microsclerotium formed in (M) egg and (N) pure culture, forming a *textura angulari* in optical sections. Material obtained from (B-G, M) infected cysts directly placed and incubated on fungal colony, (H-L) slide cultures, (N) OA. Scale bars: A = 300 μm; (B-N) = 30 μm.

*Ijuhya vitellina* infected eggs of *H*. *filipjevi in vitro*. Eggs in healthy cysts placed on colonies of *I*. *vitellina* became parasitized by hyphae within four weeks (Fig 6A). Hyphal cells inside eggs enlarged and destroyed unembryonated eggs or developing juveniles (Fig 6B). Eggs were entirely occupied by compartmentalised thick-walled hyphae (Fig 6C), and guttules-filled moniliform chlamydospores (Fig 6D) that eventually developed into microsclerotia (Fig 6E–6G). Such microsclerotia were observed within 4–5 weeks after cysts or single eggs were placed on fungal colonies; they measured 26–66 x 31–75 (41 x 49) μm (n = 97) in eggs of 3-month-old infected cysts.

Slide culture based observations revealed that individual eggs were infected within two weeks by hyphae that emerged from microsclerotia used as inoculum. Infection started with individual hyphae or appressorium-like structures that penetrated the eggshell and cuticle of developing juveniles (Fig 6H). Following penetration, similar infection processes and structures as described above, including swollen hyphal cells, thick-walled and multicellular structures filled with guttules, and subglobose or ellipsoidal microsclerotia were observed (Fig 6I–6L). Microsclerotia developing inside artificially infected eggs (Fig 6M and 6N) appearing *textura angularis* (cf. Fig 5H for details) were indistinguishable from those encountered in field-collected cysts (cf. Fig 1B and 1C).

### Metabolite profiling

#### LC-MS analysis of the crude extracts

Crude extracts obtained from Q6/2 medium showed highest antimicrobial activity. Therefore, this medium was chosen for up-scaling purposes. HPLC-UV chromatogram of the crude extract of *I*. *vitellina* revealed two major peaks at retention times 10.8 and 12.1 min. The peak at 10.8 min showed molecular ion peaks at *m/z* 529.2 [M+H]^+^, 527.2 [M-H]^-^ and 511.2 [M+H-H_2_O]^+^. Accordingly, the molecular mass of compound **1** was determined as 528.2 g/mol. Similarly, the molecular mass of compound **2** was determined as *m/z* 570.2 g/mol (S3–S5 Figs). After scale-up fermentation, the crude extract was purified as described in the experimental section and obtained pure compounds were submitted to HRESIMS and NMR analysis for structure elucidation.

#### Structure determination of chaetoglobosins

**Fig 7:**
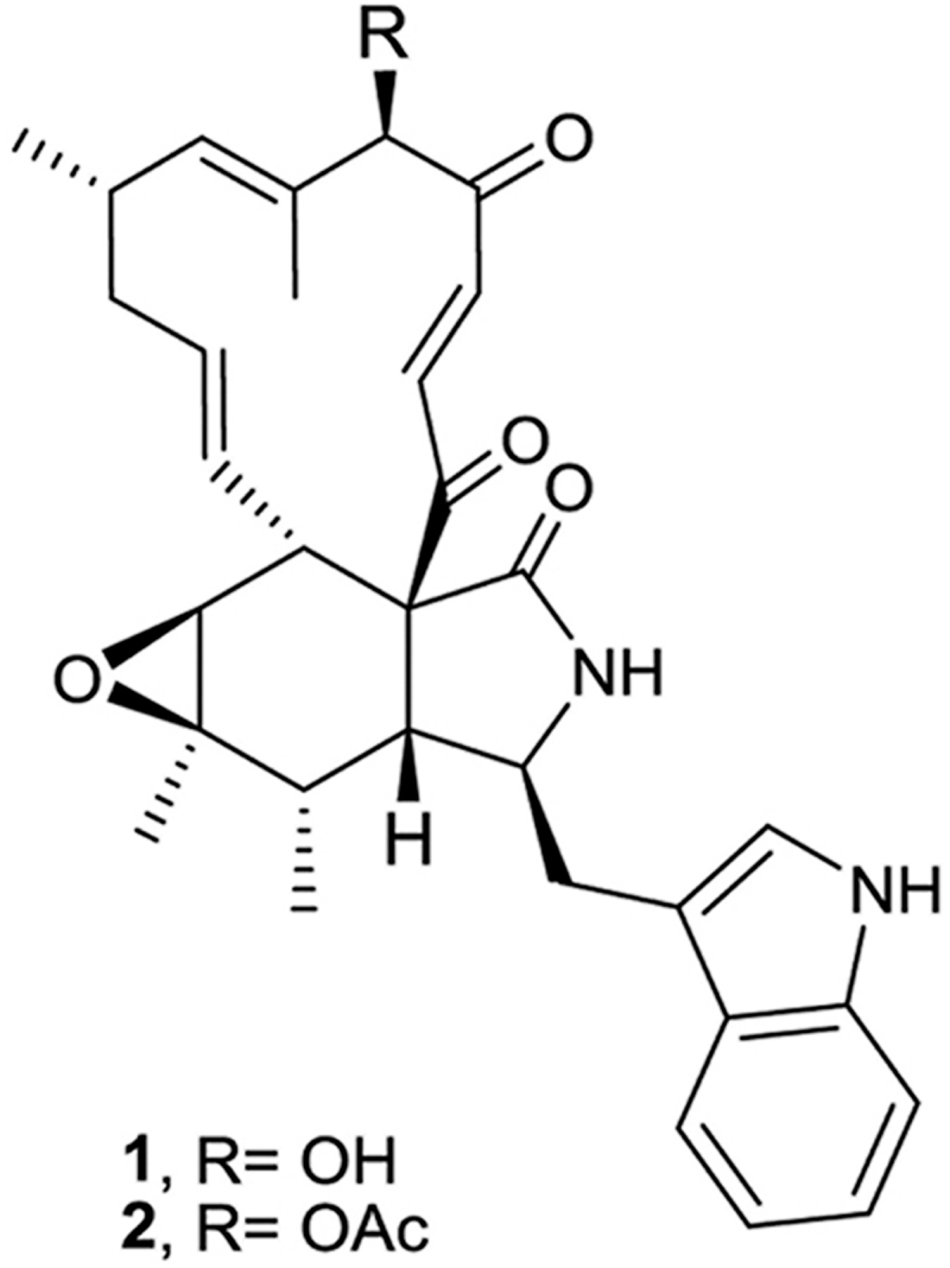
Structures of chaetoglobosin A (1) and 19-*O*-acetylchaetoglobosin A (2).

HRMS analysis of compounds **1** and **2** revealed the molecular formulae C_32_H_36_N_2_O_5_ and C_34_H_38_N_2_O_6_, respectively. Comprehensive analysis of the 1D and 2D NMR data of **1** and **2** indicated that compound **1** is chaetoglobosin A [16, 83] while compound **2** is its 19-*O*-acetylchaetoglobosin A [84] (Fig 7). The detailed description of the structure elucidation is included in the supporting information (S1 Text and S2 Text and S6 Fig).

#### Screening of Ijuhya spp. for chaetoglobosins and other secondary metabolites

Chaetoglobosin A and its derivatives could not be found in eight other *Ijuhya* species. In the range of the retention times of the isolated chaetoglobosins (ca. 10–12 min) no related chaetoglobosins with similar masses and UV/Vis spectra [85, 86] were detected.

#### Microtiter plate assay for nematicidal activities

Chaetoglobosin A and 19-*O*-acetylchaetoglobosin A caused a temporary immobilisation of *C*. *elegans* and the second stage juveniles of *H*. *filipjevi* at 50 and 100 μg/mL. The immobilisation rate was higher at 100 μg/mL and for chaetoglobosin A. Both nematode species were immobilised shortly after having been exposed to the solutions. No nematicidal activities of the tested compounds were observed.

#### Cytotoxicity bioassay

Chaetoglobosin A (**1**) inhibited cell lines L929, KB3.1, PC-3 and HUVEC with IC_50_ values in μg/mL of 1.6, 0.15, 0.42 and 0.78, respectively. The acetyl derivative (**2**) showed inhibition against the same cell lines with IC_50_ values of 0.7, 0.19, 0.7 and 0.25 μg/mL, respectively.

## Discussion

### Phylogenetic analyses and systematic implications

*Ijuhya vitellina* is inferred as a new species on the basis of comparative morphological and molecular phylogenetic evidences. Phylogenetically, and supported by DNA sequences of five gene regions, the fungus occupies a distinct and highly supported monophyletic species clade nesting in the *Ijuhya* core group of the Bionectriaceae. Classification of the new species in genus *Ijuhya* is, however, purely based on phylogenetic evidence. While all other *Ijuhya* species are teleomorphically typified and largely characterized by morphological characters of ascomata and ascospores [82, 87, 88], the teleomorph of *I*. *vitellina* is unknown. Nematode associated life-style has never been described for *Ijuhya* species before. Other members of the genus have so far been found on plant substrata. *Ijuhya vitellina* differs most clearly from other *Ijuhya* species by the formation of brightly coloured, orange to reddish microsclerotia that have not been described for any of the other *Ijuhya* species. Further phylogenetic analyses on the basis of alternative taxon selections and additional data are thus required to confirm monophyly of *I*. *vitellina* with *Ijuhya* or resolve *I*. *vitellina* outside *Ijuhya sensu stricto* rendering the description of an additional genus necessary. *Ijuhya vitellina*, based on our taxon sampling, is most closely related to *I*. *corynospora* described from dead leaves of *Phormium tenax* in New Zealand (Fig 2). However, also in this case no correlating characters supporting this sister group relationship can be found. Neither were chlamydospores or microsclerotia described for *I*. *corynospora sensu stricto* [89] nor did it parasitise nematode eggs in our *in vitro* experiments. Also *I*. *vitellina* does form chaetoglobosins, while no such metabolites were encountered in *I*. *corynospora* or in any other of the here studied *Ijuhya* species. Phylogenetic analyses suggest that *I*. *antillana*, *I*. *dentifera*, and *I*. *oenanthicola* are only distantly related to *Ijuhya sensu stricto* and not part of the genus. Morphological characters described for *I*. *antillana* [90] and *I*. *oenanthicola* [91] conform well to the concept of the *Nectria sylvana* group [87], for which *Lasionectria* became the generic depository [82]. Specifically, fasciculate hyphal clusters formed by perithecia of *I*. *antillana* (Fig 1A, 1B and 1D in Lechat and Courtecuisse [90]) and *I*. *oenanthicola* (Plate 1, Fig. A–C in Lechat and Hairaud [91]) and occurrence of 1-septate striate ascospores are similar to those seen in *Lasionectria sensu stricto*. Accordingly, *I*. *antillana* and *I*. *oenanthicola* are combined into *Lasionectria* (Appendix).

### Parasitism on nematode cysts and eggs

Our observations from field collected nematode cysts and various *in vitro* infection studies showed that *I*. *vitellina* parasitises cereal cyst nematodes. Several ascomyceteous fungal species have been reported to parasitise plant parasitic nematodes including cyst nematodes [2, 92–94]. Within the Bionectriaceae, to our knowledge only two species, *Clonostachys rosea* and *Gliomastix murorum*, were described as antagonists of animal- and plant parasitic nematodes [7, 95, 96].

Hyphae of *I*. *vitellina* penetrate nematode eggs either directly or by developing an appressorium-like structure. We purport that hyphae of *I*. *vitellina* may similarly enter nematode cysts or juveniles. Upon penetration the fungus forms hyphae inside eggs as is also reported for other cyst nematode parasitic fungi [2]. Penetration may apply through mechanic or chemical mechanisms [97–100]. Interactions involving chemical mechanisms are based on enzymes whose activities allow the penetration of the multilayered eggshell that in cyst nematodes mainly consists of chitin and lipids [2, 101–104]. Hyphal penetrations by *I*. *vitellina* may thus involve similar strategies to invade eggs, juveniles and cysts of *H*. *filipjevi*.

Hyphae coiling around and penetrating nematode eggs and filling the content of eggs have been described previously in various cases [105]. It is possible that hyphae directly use nutritional resources provided by nematode eggs for hyphal growth and mycelium development. The behaviour of *I*. *vitellina* inside nematode eggs differs drastically. Hyphae immediately swell and become transformed into globose or ellipsoidal dictyochlamydospores that develop into microsclerotia. Cells in these structures are angular and filled with guttules. Microsclerotia are rigid and readily resist mechanical manipulations. Additionally, guttules forming inside microsclerotia could contain lipid-like compounds for the storage of energy or may provide protection against desiccation [106]. Thus, these structures might play an important role in the survival of *I*. *vitellina*, for example during drought stress or other harsh environmental conditions. In addition, *I*. *vitellina* may not only recruit nutrients from nematode eggs for hyphal development, but inhabit these eggs inside cysts for protection and long-time survival. This is a plausible explanation as also healthy eggs of *H*. *filipjevi* can survive several years inside cysts. Empirical support for this hypothesis comes from the observation that we were (still) able to isolate *I*. *vitellina* from infected eggs after field-collected cysts were kept for several months at 4°C. The formation of microsclerotia in *I*. *vitellina* could be considered as the start and the end of the fungus’ development, at least with respect to those parts of its life cycle that were studied here. Once formed within nematode eggs, microsclerotia may remain inactive but produce newly emerging hyphae when the life-cycle of this species is newly initiated under favourable environmental conditions.

Guttules similar to those formed by *I*. *vitellina* have been suggested to serve as energy reservoirs in some other nematode parasitic fungi, *e*.*g*., the trap-forming *Arthrobotrys* species [107] and could also be involved in the parasitism of nematodes [105]. It was reported that guttules may contain linoleic acid as a compound responsible for nematode killing [13]. Dijksterhuis et al. [108] also suggested that microbodies, *e*.*g*., lipid organelles, present infection-related features in nematophagous fungi. However, their exact functions have not been fully elucidated. This is the first time that fungal survival structures were encountered inside nematode eggs and, accordingly, a new mode of fungus nematode interaction is described herewith.

Cysts provide a protected environment for nematode eggs where biotic and abiotic stresses are significantly reduced and eggs survive several years. Such niche might thus be a suitable environment for a nematophagous fungus where it may produce equally long-living survival structures. This situation, along with the presence of mucilaginous content of cysts, might even accelerate the fungal growth and provide optimum conditions for the fungus to colonise the entire cyst cavity and parasitise the eggs. In all cysts collected in fields, however, only a fraction of cysts carried eggs infected with microsclerotia. It is possible that *I*. *vitellina* survives in a dormant stage as microsclerotia inside cysts and that it emerges from individually parasitised eggs at favorable conditions, *e*.*g*., at times juveniles hatch from non-infected eggs within the same cyst. Microsclerotia encountered in culture have similar shapes and sizes as those *I*. *vitellina* forms in nematode eggs, either *in vitro* or in field collected cysts. The same applies for the cells of these microsclerotia. Accordingly this suggests that in field and *in vitro* encountered microsclerotia are homologous. If formed in culture they may therefore mimic the egg-parasitising habit of *I*. *vitellina* in nature. Absence of such microsclerotia in other closely related species of *Ijuhya* could therefore suggest that *I*. *vitellina* is the only nematode parasitising species of this genus.

### Metabolite profiling

*Ijuhya vitellina* is reported here as a novel source of chaetoglobosin A. The vast majority of chaetoglobosins (A, B, C, D, E, F, G, and J) and their respective derivatives have mostly been isolated from the fungus *Chaetomium globosum* [15, 16, 109, 110]. Chaetoglobosin C is also produced by *Penicillium aurantiovirens* [111], and chaetoglobosin K was first extracted from *Diplodia macrospora* [18]. Interestingly, no such chaetoglobosins were encountered in the other, closely related *Ijuhya* species including *I*. *chilensis*, *I*. *corynospora*, *I*. *faveliana*, *I*. *parilis*, and *I*. *peristomialis*. Whether chaetoglobosin A and its acetyl derivative play a role in nematode egg parasitism can be inferred only with uncertainty. A temporary inhibition of mobility was observed when the two chaetoglobosins were tested *in vitro* against *C*. *elegans* and *H*. *filipjevi*. Chaetoglobosins affect and inhibit polymerization of actin and can degrade microfilaments [17, 112]. This might explain our observation of the effect of chaetoglobosin A and its derivative 19-*O*-acetylchaetoglobosin A on paralyzing the tested nematodes at 50 and 100 μg/mL. However, at higher concentrations (300 μg/mL) chaetoglobosin A was reported to have toxic effects and caused nematode mortality [26]. Thus, chaetoglobosins produced by *I*. *vitellina* may have a function in the described parasitism of nematode eggs.

## Appendix

*Lasionectria* (Sacc.) Cooke, Grevillea 12: 111. 1884.

Holomorphs of species described in *Lasionectria* are characterized by perithecia often showing triangular fascicles of densely packed hyphae that emerge from outer perithecial wall regions and 1-septate ascospores [82]. Structures illustrated for *Ijuhya antillana* [90] and *Ijuhya oenanthicola* [91] are conform with the generic concept of *Lasionectria*. Both species are phylogenetically closely related with *Lasionectria mantuana* (S1 and S2 Figs), which is the type species of genus *Lasioinectria*. Accordingly the following combinations are suggested:

*Lasionectria antillana* (Lechat & Courtec.) Schroers, Ashrafi, W. Maier comb. nov., Mycobank MB 821498. Basionym, *Ijuhya antillana* Lechat & Courtec., Mycotaxon 113: 444. 2010. Mycobank MB516744.

*Lasionectria oenanthicola* (Lechat & Hairaud) Schroers, Ashrafi, W. Maier comb. nov., Mycobank MB 821499. Basionym, *Ijuhya oenanthicola* Lechat & Hairaud, Mycotaxon 119: 249. 2012. Mycobank MB561714.

## Supporting information

S1 Fig Bayesian inference of phylogenetic relationships of selected taxa of the Bionectriaceae and Nectriaceae (Hypocreales) based on LSU and *rpb1* sequences. Numbers above nodes are estimates of *a posteriori* probabilities greater than 0.94 / NJB and MLB values greater than 70%. The topology was rooted with *Aschersonia placenta*, *Balansia henningsiana*, *B*. *pilulaeformis*, and *Moelleriella libera* (Hypocreales). Two highly supported subclades are suggested for the in-group of genus *Ijuhya*, of which one includes *I*. *peristomialis*, *I*. *chilensis*, *I*. *faveliana*, *I*. *paraparilis*, and *I*. *parilis*. The other subclade includes *I*. *vitellina* and its closest sister species, *I*. *corynospora*. The distantly related *I*. *antillana* and *I*. *oenanthicola* are inferred as phylogenetic relatives of *Lasionectria mantuana*.
(TIF)

S2 Fig Bayesian inference of phylogenetic relationships of selected taxa of the Bionectriaceae and Nectriaceae (Hypocreales) based on *act*, LSU, and *rpb1* sequences. Numbers above nodes are estimates of *a posteriori* probabilities greater than 0.94 / NJB and MLB values greater than 70%. The topology was rooted with *Aschersonia placenta*, *Balansia henningsiana*, *B*. *pilulaeformis*, and *Moelleriella libera* (Hypocreales). Two highly supported subclades are suggested for the in-group of genus *Ijuhya*, of which one includes *I*. *peristomialis*, *I*. *chilensis*, *I*. *faveliana*, *I*. *paraparilis*, and *I*. *parilis*. The other subclade includes *I*. *vitellina* and its closest sister species, *I*. *corynospora*. The distantly related *I*. *antillana* and *I*. *oenanthicola* are inferred as phylogenetic relatives of *Lasionectria mantuana*.
(TIF)

S3 Fig LCMS Chromatogram for the crude extract of *Ijuhya vitellina*. Peaks represent chaetoglobosin A (1) and 19-*O*-acetylchaetoglobosin A (2); Insertion is the UV-VIS spectrum of chaetoglobosin A (1).
(TIF)

S4 Fig Mass spectrum of chaetoglobosin A (1). (TIF)

S5 Fig Mass spectrum of 19-*O*-acetylchaetoglobosin A (2). (TIF)

S6 Fig 2D NMR assignment of chaetoglobosin A (1) and 19-*O*-acetylchaetoglobosin A (2). HMBC (arrows) and COSY (bold bonds) correlations.
(TIF)

S1 Text Structure determination of chaetoglobosins. (PDF)

S2 Text Spectroscopic data for chaetoglobosin A (1) and 19-*O*-acetylchaetoglobosin A (2). (PDF)

S1 Table NMR spectroscopic data for chaetoglobosin A (1). (PDF)
