# Supplementary figures and images for: Ijuhya vitellina sp. nov., a novel source for chaetoglobosin A, is a destructive parasite of the cereal cyst nematode Heterodera filipjevi
Source: PLoS One. 2017 Jul 12;12(7):e0180032. doi: 10.1371/journal.pone.0180032 (PMC5507501; doi:10.1371/journal.pone.0180032)

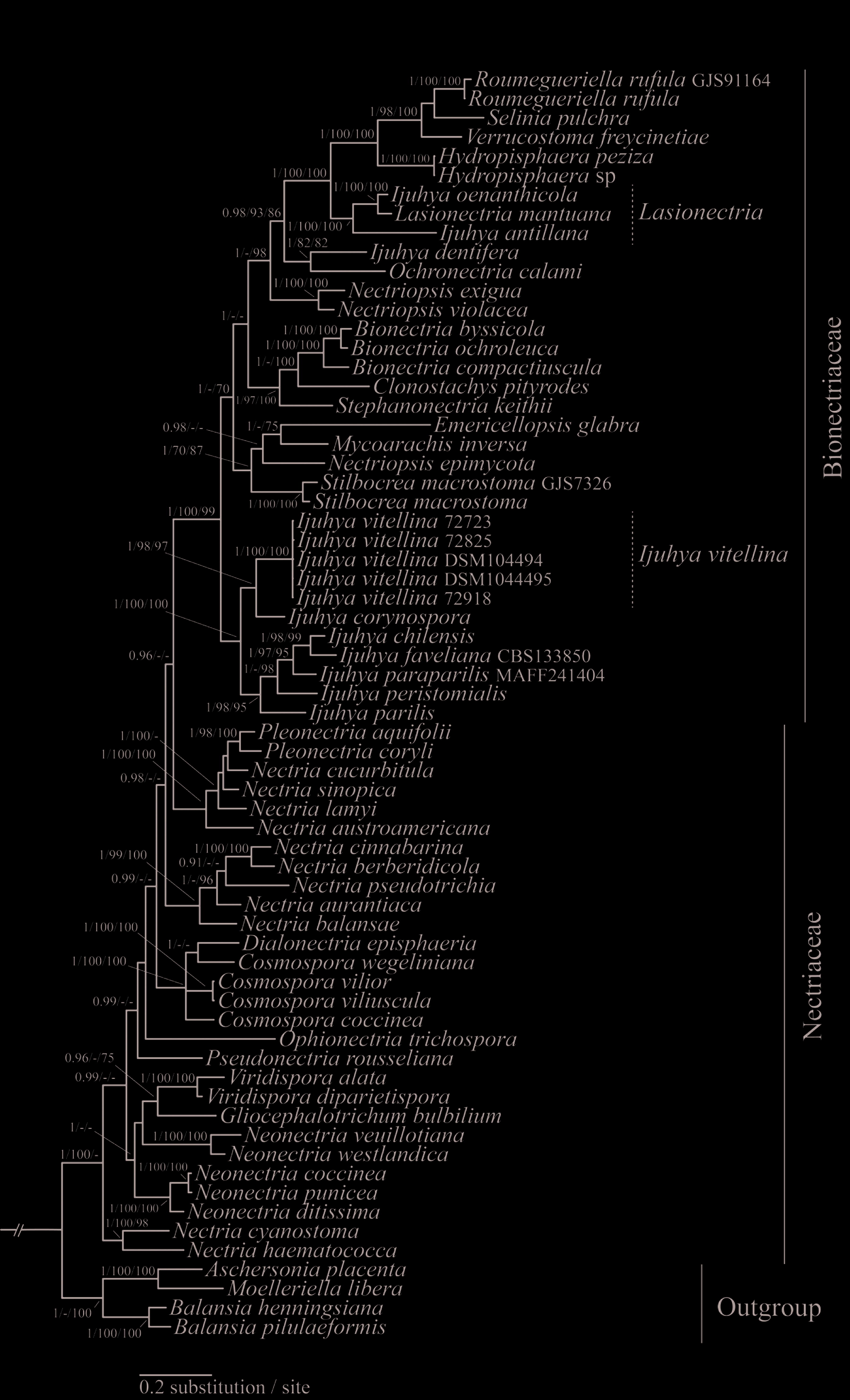

Supplement: S1 Fig — Numbers above nodes are estimates of a posteriori probabilities greater than 0.94 / NJB and MLB values greater than 70%. The topology was rooted with Aschersonia placenta, Balansia henningsiana, B. pilulaeformis, and Moelleriella libera (Hypocreales). Two highly supported subclades are suggested for the in-group of genus Ijuhya, of which one includes I. peristomialis, I. chilensis, I. faveliana, I. paraparilis, and I. parilis. The other subclade includes I. vitellina and its closest sister species, I. corynospora. The distantly related I. antillana and I. oenanthicola are inferred as phylogenetic relatives of Lasionectria mantuana. (TIF) [file pone.0180032.s001.tif]

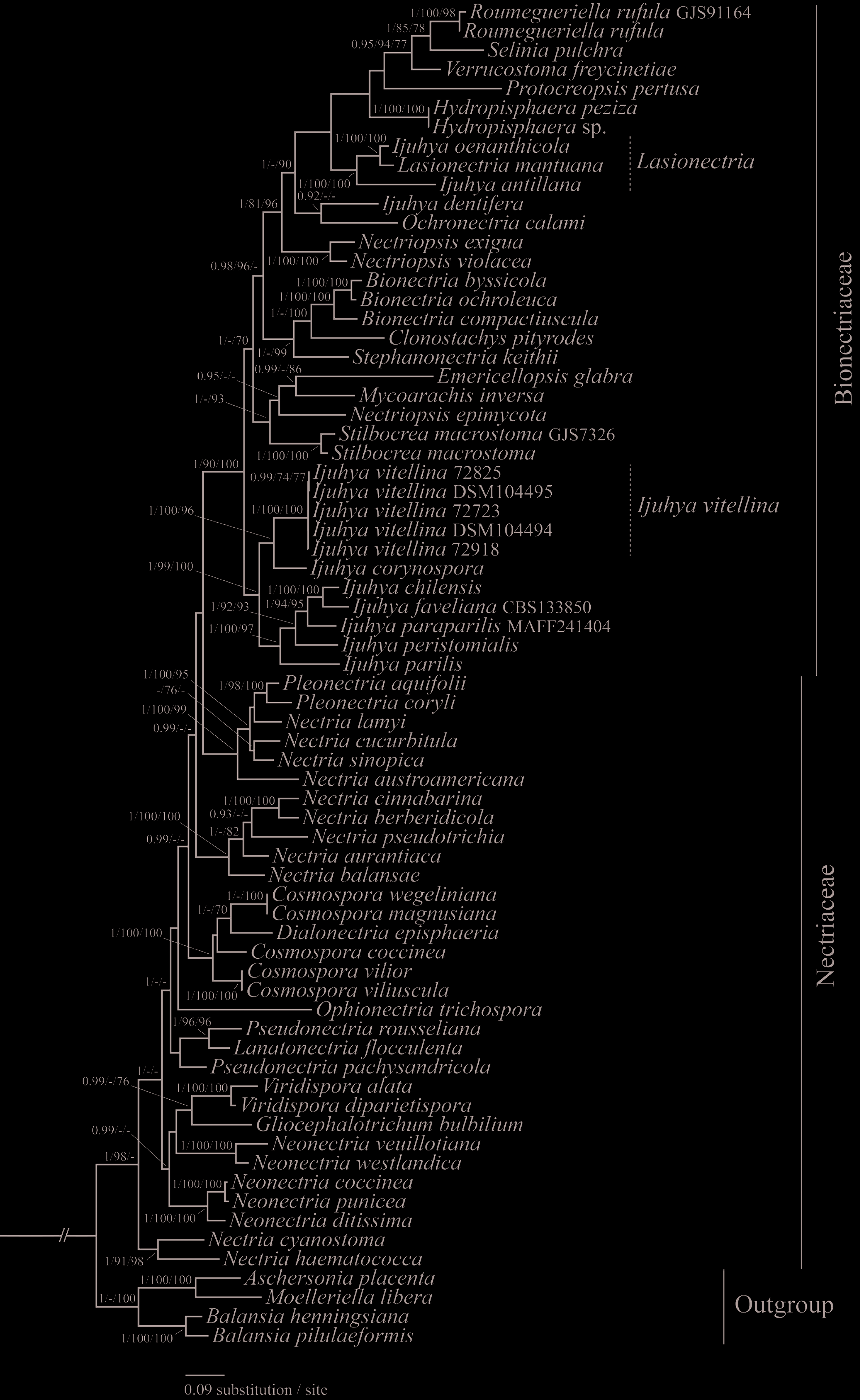

Supplement: S2 Fig — Numbers above nodes are estimates of a posteriori probabilities greater than 0.94 / NJB and MLB values greater than 70%. The topology was rooted with Aschersonia placenta, Balansia henningsiana, B. pilulaeformis, and Moelleriella libera (Hypocreales). Two highly supported subclades are suggested for the in-group of genus Ijuhya, of which one includes I. peristomialis, I. chilensis, I. faveliana, I. paraparilis, and I. parilis. The other subclade includes I. vitellina and its closest sister species, I. corynospora. The distantly related I. antillana and I. oenanthicola are inferred as phylogenetic relatives of Lasionectria mantuana. (TIF) [file pone.0180032.s002.tif]

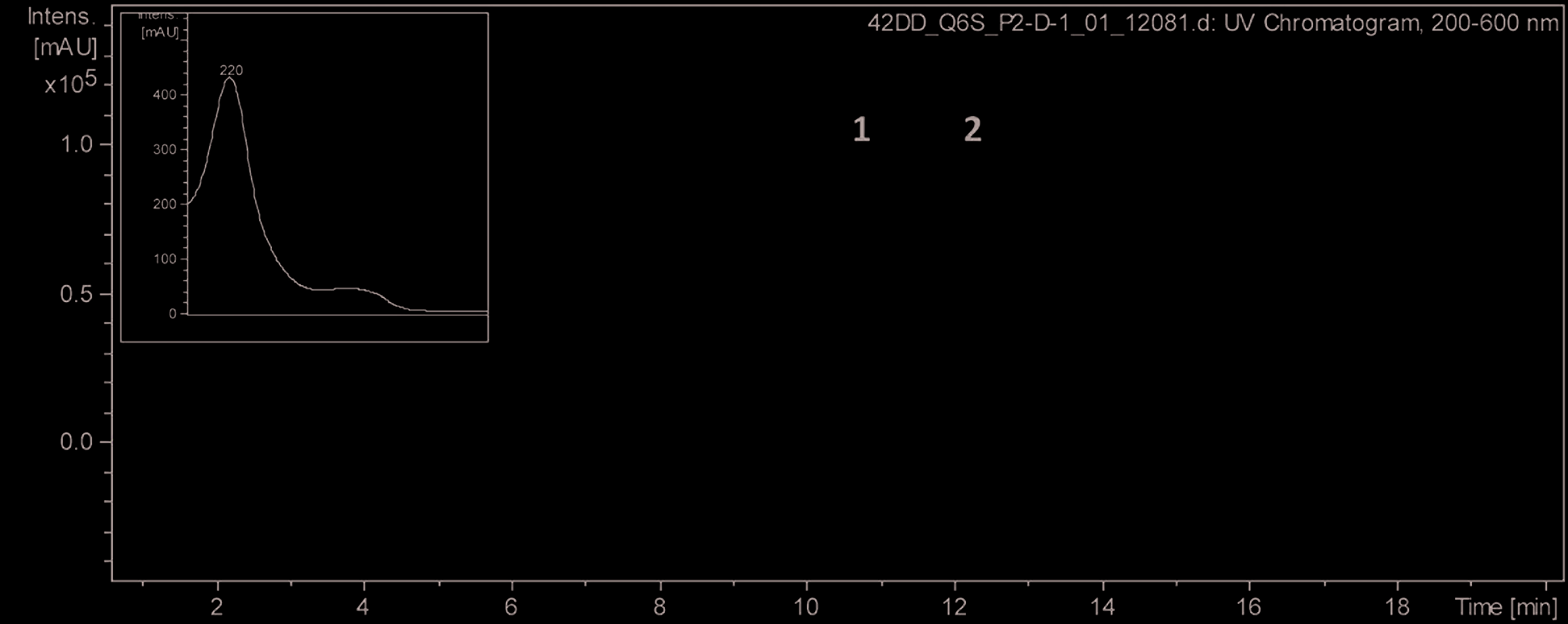

Supplement: S3 Fig — Peaks represent chaetoglobosin A (1) and 19-O-acetylchaetoglobosin A (2); Insertion is the UV-VIS spectrum of chaetoglobosin A (1). (TIF) [file pone.0180032.s003.tif]

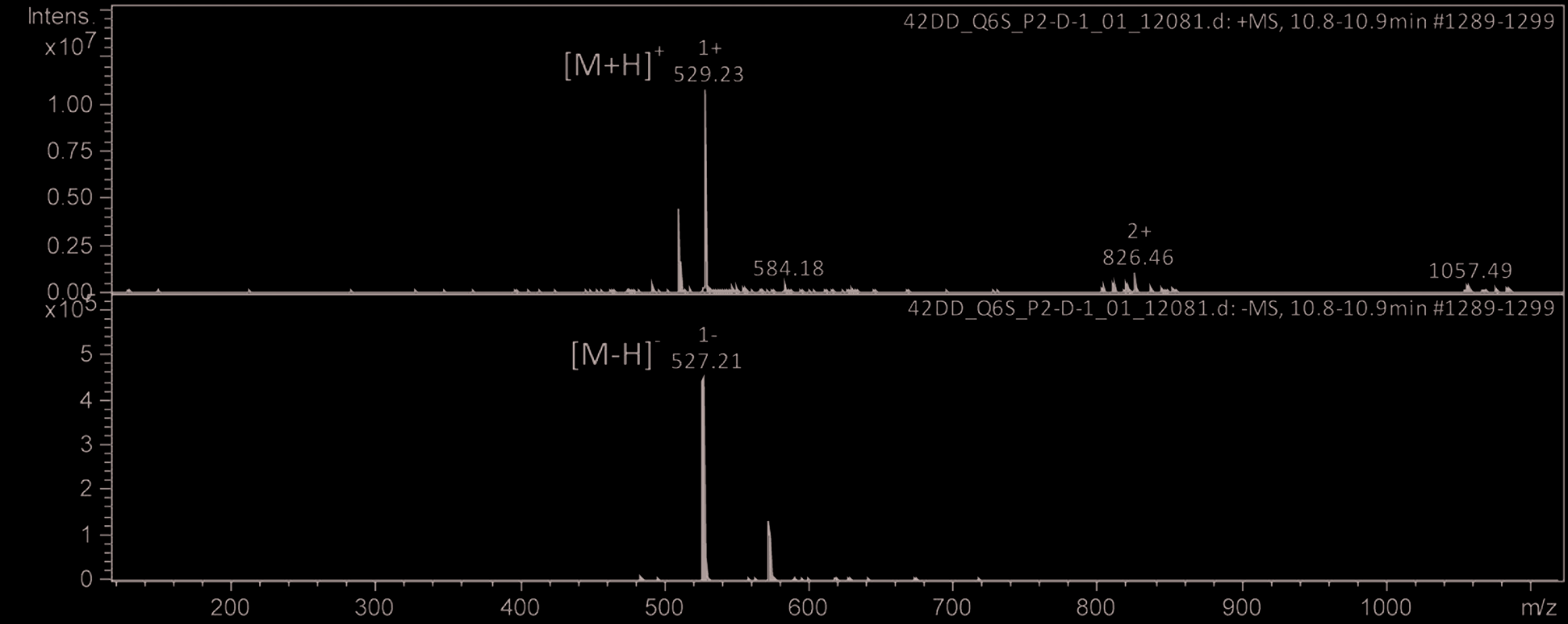

Supplement: S4 Fig — (TIF) [file pone.0180032.s004.tif]

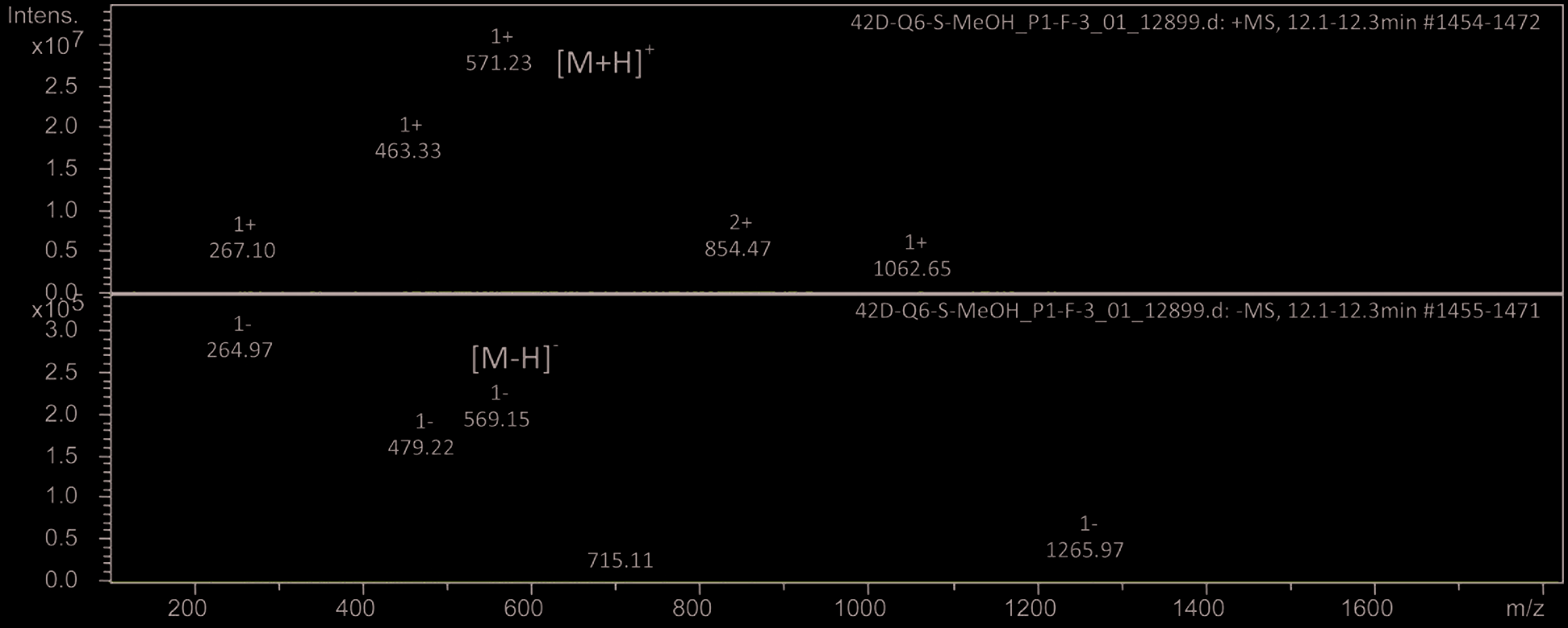

Supplement: S5 Fig — (TIF) [file pone.0180032.s005.tif]

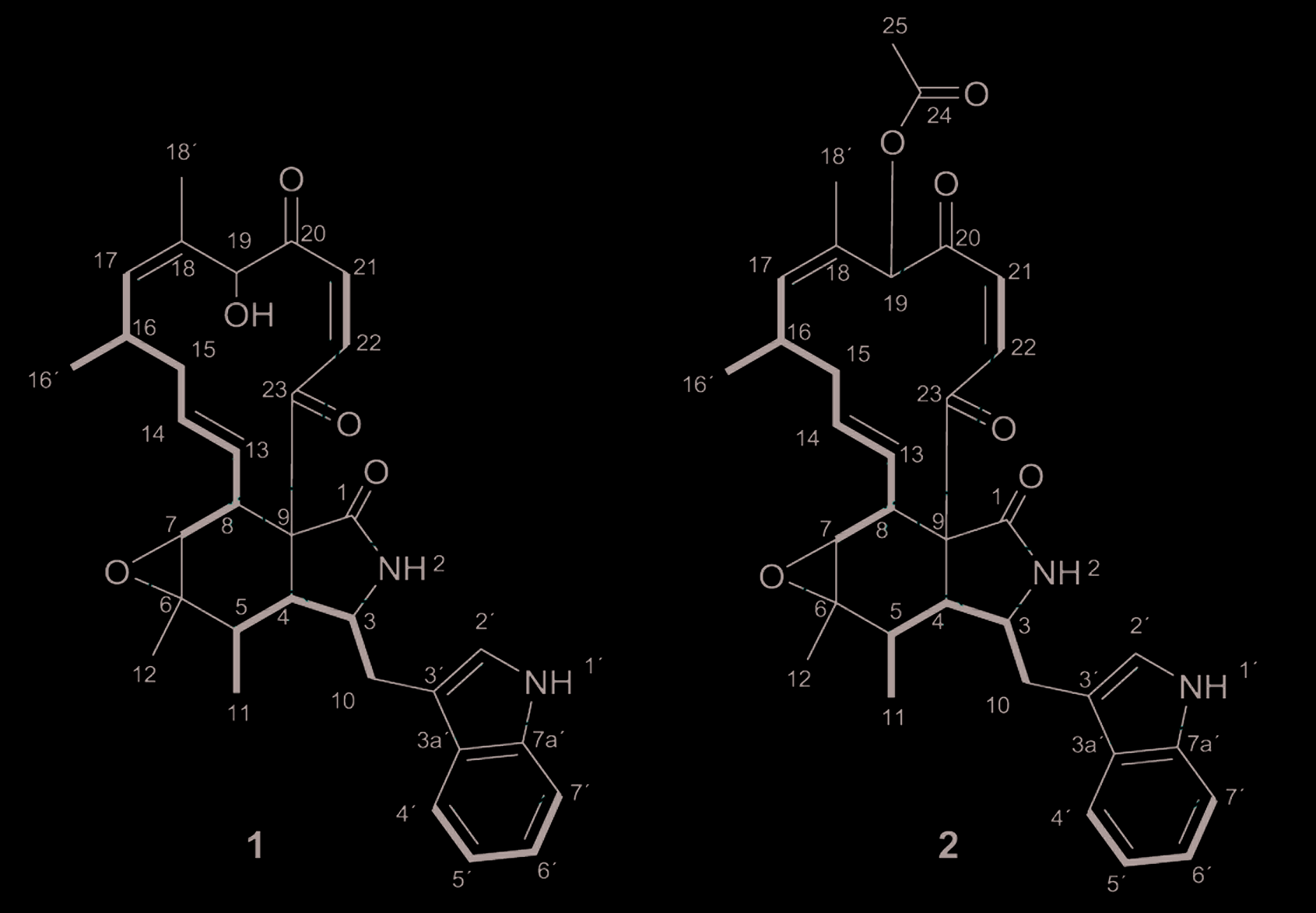

Supplement: S6 Fig — HMBC (arrows) and COSY (bold bonds) correlations. (TIF) [file pone.0180032.s006.tif]
