# Supplementary material for: Ijuhya vitellina sp. nov., a novel source for chaetoglobosin A, is a destructive parasite of the cereal cyst nematode Heterodera filipjevi
Source: PLoS One. 2017 Jul 12;12(7):e0180032. doi: 10.1371/journal.pone.0180032 (PMC5507501; doi:10.1371/journal.pone.0180032)
Supplement: S1 Text — (PDF) [file pone.0180032.s007.pdf]

S1 Text. Structure determination of chaetoglobosins

Compound **1** was obtained as yellow powder. Its molecular formula was determined as $C_{32}H_{36}N_2O_5$ by the HRESIMS which showed ion peak at *m/z* 529.2699 $[M+H]^+$ (calcd 529.2697). The ^{1}H NMR spectrum of **1** exhibited signals for 10 aromatic/olefinic protons, seven methines, two methylens and four methyls. Two additional signals were assigned to hetero protons suggested the presence of two NH and/or OH groups in **1**. The ^{13}C NMR showed 32 carbon resonances, of which 23 were observed in DEPT spectrum indicating 9 non-protonated carbons among them 3 carbonyls (δ_C 201.7, 196.7 and 172.9) (S1 Table). The planar structure of compound **1** was deduced from the 2D NMR data including COSY and HMBC spectra. COSY correlation between H-4´/H-5´/H-6´/H-7´ together with HMBC correlations from H-4´to C-6´/C-7a´/C-3´; H-7´ to C-5´/C-3a´; H-2´ to C-3a´/C-7a´ and from NH-1´ to C-2´/C-3´/C-3a´/C-7a´ revealed the presence of an indole moiety in compound **1** (S6 Fig). In addition, an isoindole derivative was assigned by HMBC correlations network from NH-2 to C-1/C-3/C-4/C-9; a group of HMBC correlations from H-4 to C-1/C-3/C-5/C-6/C-8/C-9; H_3-10 to C-4/C-5/C-6; H_3-12 to C-5/C-6/C-7; COSY correlation between H-7 and H-8 together with HMBC correlations from H-7 to C-6/C-9 and from H-8 to C-7/C-9/C-1 allowed the construction of 6,6a-dimethylhexahydro-1a*H*-oxireno[2,3-f]isoindol-3(4*H*)-one. The above mentioned structure units of compound **1** was connected by methylene group assigned by means of COSY correlation from H-3 to H-10 and a series of HMBC correlations from H-10 to C-3/C-4/C-2´/C-3´/C-4´ as well as correlations from H-2´ to C-10. Finally, a chain of 11-carbon atoms was determined in the same manner by COSY and HMBC correlations, whereas COSY correlations were determined for a series of protons from H-13 to H-17 and HMBC correlations from H_3-16´ to C-15/C-16/C-17; from H_3-18´ to C-17/C-18/C-19. Furthermore, COSY correlation between the two olefinic protons H-21 and H-22 with HMBC from H-21 to the carbonyl carbon C-23 and to C-9 and from H-4 to C-23; H-7 to C-14, H-13 to C-7/C-8 allowed the construction of 13-membered ring at C-8/C-9 of the isoindole moiety.

Compound **2** was isolated as a yellow powder. It has the molecular formula of $C_{34}H_{38}N_2O_6$ deduced from the HRESIMS data that showed a peak at *m/z* 571. 2803 $[M+H]^+$ (calcd 571.2803). Its UV spectrum was similar to that of chaetoglobosin A and the molecular mass difference of 42 Da

suggested that **2** was likely an acetyl derivative of **1**. The ^{1}H and ^{13}C NMR spectra of **2** were very similar to those of **1**, but the ^{13}C NMR spectrum showed two additional carbon resonances at δ_C 170.2 and δ_C 20.8, further supporting the presence of an additional acetyl group. The presence of the acetyl group was determined by HMBC correlation from H_3-25 (δ_H 2.18) to the carbonyl carbon C-24 and its position was determined by the HMBC correlation from H-19 (δ_H 5.91, δ_C 83.3) to C-24.
