# Supplementary material for: Ijuhya vitellina sp. nov., a novel source for chaetoglobosin A, is a destructive parasite of the cereal cyst nematode Heterodera filipjevi
Source: PLoS One. 2017 Jul 12;12(7):e0180032. doi: 10.1371/journal.pone.0180032 (PMC5507501; doi:10.1371/journal.pone.0180032)
Supplement: S2 Text — (PDF) [file pone.0180032.s008.pdf]

S2 Text. Spectroscopic data for chaetoglobosin A (1) and 19-*O*-acetylchaetoglobosin A (2)

Chaetoglobosin A (1):

Yellow powder; $[\alpha]^{20}_{D}$ - 165° (*c* 0.1, MeOH); NMR data see Table S1; MS data *m/z* 529.21 $[M+H]^+$, 511.22 $[M+H-H_2O]^+$, 527.20 $[M-H]^-$, 573.15 $[M-H+HCOOH]^-$; HRESIMS *m/z* 529.2699 $[M+H]^+$ (calcd 529.2697 for $C_{32}H_{37}N_2O_5$).

19-*O*-acetylchaetoglobosin A (2):

Yellow powder; $[\alpha]^{25}_{D}$ - 147° (*c* 0.1, MeOH); ^1H NMR (700 MHz, CDCl3) δ_H 1.02 (d, *J*=6.88 Hz, H-16´), 1.25 (d, *J*=7.31 Hz, H-11), 1.30 (s, H-12), 1.47 (d, *J*=1.29 Hz, H-18´), 1.86 (m, H-5), 2.05 (dt, *J*=13.77, 10.97 Hz, H-15a), 2.15 (dd, *J*=9.68, 4.95 Hz, H-8), 2.20 (s, H-25), 2.27 (m, H-15b), 2.46 (m, H-16), 2.67 (dd, *J*=14.63, 7.74 Hz, H-10a), 2.80 (d, *J*=5.16 Hz, H-7), 2.91 (m, H-10b), 2.93 (m, H-4), 3.80 (dt, *J*=7.21, 3.50 Hz, H-3), 5.20 (ddd, *J*=15.06, 10.76, 3.87 Hz, H-14), 5.71 (dd, *J*=9.03, 1.29 Hz, H-17), 5.74 (s, NH-2), 5.93 (s, H-19), 6.09 (ddd, *J*=15.17, 9.79, 1.29 Hz, H-21), 6.39 (br d, *J*=16.78 Hz, H-22), 7.01 (d, *J*=2.15 Hz, H-2´), 7.15 (dd, *J*=6.88, 7.31 Hz, H-5´), 7.21 (dd, *J*=6.88, 8.17 Hz, H-6´) 7.37 (d, *J*=8.17 Hz, H-7´), 7.50 (d, *J*=7.74 Hz, H-4´), 7.58 (d, *J*=16.78 Hz, H-22), 8.20 (br s, NH-1´); ^{13}C NMR (176MHz, CHLOROFORM-d) δ = 196.8 (C, C-23), 195.0 (C, C-20), 173.3 (C, C-1), 170.1 (C, C-24), 142.6 (CH, C-17), 136.3 (C, C-7a´), 134.8 (CH, C-22), 133.4 (CH, C-14), 133.2 (CH, C-21), 128.3 (CH, C-13), 127.9 (C, C-18), 127.0 (C, C-3a´), 123.5 (CH, C-2´), 122.6 (CH, C-5´), 120.1 (CH, C-6´), 118.3 (CH, C-4´), 111.7 (CH, C-7´), 110.2 (C, C-3´), 83.3 (CH, C-19), 63.2 (C, C-9), 62.3 (C, C-7), 57.9 (C, C-6), 52.4 (C, C-3), 48.6 (CH, C-8), 47.4 (CH, C-4), 41.5 (CH_2, C-14), 36.3 (CH, C-5), 34.1 (CH_2, C-10), 32.2 (CH, C-16), 20.84 (CH_3, C-25), 20.81 (CH_3, C-16´), 19.9 (CH_3, C-12), 13.5 (CH_3, C-11), 11.7 (CH_3, C-18´); MS data *m/z* 571.22 $[M+H]^+$, 511.22 $[M+H-AcOH]^+$, 569.15 $[M-H]^-$, 615.12 $[M-H+HCOOH]^-$; HRESIMS *m/z* 571.2803 $[M+H]^+$ (calcd 571.2803 for $C_{34}H_{39}N_2O_5$).
