# Supplementary material for: Ijuhya vitellina sp. nov., a novel source for chaetoglobosin A, is a destructive parasite of the cereal cyst nematode Heterodera filipjevi
Source: PLoS One. 2017 Jul 12;12(7):e0180032. doi: 10.1371/journal.pone.0180032 (PMC5507501; doi:10.1371/journal.pone.0180032)
Supplement: S1 Table — (PDF) [file pone.0180032.s009.pdf]

**S1 Table. NMR spectroscopic data for chaetoglobosin A (1) (700 MHz, $CDCl_3$)**

| Pos. | **1** | | |
|---|---|---|---|
| | $\delta_C$, type | $\delta_H$, mult. ( *J* in Hz) | HMBC |
| 1 | 172.9, C | - | - |
| 2 | - | 5.73, s | 1, 9, 3, 4 |
| 3 | 52.6, CH | 3.79, m | 1, 4, 5 |
| 4 | 47.2, CH | 3.05, dd (4.73, 3.44) | 1, 3, 5, 6, 8, 9, 23 |
| 5 | 36.2, CH | 1.86, m | 3, 4, 6, 9, 11, 12 |
| 6 | 58.0, C | - | - |
| 7 | 62.4, CH | 2.81, d (4.73) | 6, 8, 9, 12, 13 |
| 8 | 48.9, CH | 2.15, dd (9.90, 4.73) | 7, 9, 1, 13, 14 |
| 9 | 63.2, C | - | - |
| 10 | 34.4, $CH_2$ | 2.63, dd (14.63, 8.17) | |
| | | 2.97, dd (14.63, 3.87) | 2´, 3´, 3a´, 3, 4 |
| 11 | 13.6, $CH_3$ | 1.27, d (7.31) | 4, 5, 6 |
| 12 | 19.8, $CH_3$ | 1.31, s | 5, 6, 7 |
| 13 | 128.2, CH | 6.07, dd (15.27, 9.90) | 7, 8, 15 |
| 14 | 133.8, CH | 5.24, ddd (15.17, 10.86, 4.09) | 8, 15, 16 |
| 15 | 41.8, $CH_2$ | 2.05, dd (11.35, 13.44) | |
| | | 2.28, m | 13, 14, 16, 16´, 17 |
| 16 | 32.1, $CH_2$ | 2.47, m | 15, 16´, 17, 18 |
| 16´ | 21.0, $CH_3$ | 1.02, d (6.9) | 15, 16, 17 |
| 17 | 140.4, CH | 5.63, dd (9.03, 1.29) | 15, 16, 18, 19 |
| 18 | 132.3, C | - | - |
| 18´ | 10.6, $CH_3$ | 1.34, d (1.29) | 17, 18, 19 |
| 19 | 81.8, CH | 5.06, s | 17, 18´, 20, 21 |
| 20 | 201.7, C | - | - |
| 21 | 131.6, CH | 6.51, br d (16.78) | 19, 21, 22 |
| 22 | 136.4, CH | 7.77, br d (16.53) | 20, 21, 22, 9 |
| 23 | 196.7, C | - | - |
| 1´ | - | 8.14, br s | 2´, 3´, 3a´, 7a´ |
| 2´ | 123.2, CH | 6.98, d (2.15) | 3´, 3a´, 7a´, 10 |
| 3´ | 110.4, C | - | - |
| 3a´ | 126.8, C | - | - |
| 4´ | 118.3, CH | 7.50, d (7.74) | 6´, 7a´, 3´ |
| 5´ | 120.0, CH | 7.16, dd (7.42, 6.88) | 3a´, 7´ |
| 6´ | 122.5, CH | 7.22, dd (7.64, 6.88) | 4´, 7a´ |
| 7´ | 111.6, CH | 7.38, d (8.17) | 5´, 3a´ |
| 7a´ | 136.3, C | - | - |
